# Dry age-related macular degeneration like pathology in aged 5XFAD mice: Ultrastructure and microarray analysis

**DOI:** 10.18632/oncotarget.16967

**Published:** 2017-04-08

**Authors:** Sung Wook Park, Sora Im, Hyoung Oh Jun, Kihwang Lee, Young-Jun Park, Jin Hyoung Kim, Woo Jin Park, Young-Hoon Lee, Jeong Hun Kim

**Affiliations:** ^1^ Department of Biomedical Sciences, Seoul National University College of Medicine, Daehak-Ro, Jongno-Gu, Seoul, Republic of Korea; ^2^ Fight Against Angiogenesis-Related Blindness Laboratory, Biomedical Research Institute, Seoul National University Hospital, Daehak-Ro, Jongno-Gu, Seoul, Republic of Korea; ^3^ Department of Life Sciences, Life Sciences Concentration Gwangju Institute of Science and Technology Cheomdan-Gwagiro, Buk-Gu, Gwangju, Republic of Korea; ^4^ Department of Ophthalmology, Ajou University School of Medicine, World Cup-Ro, Yeongtong-Gu, Suwon-Si, Gyeonggi-Do, Republic of Korea; ^5^ Immunotherapy Research Center, Korea Research Institute of Bioscience and Biotechnology, Daejeon, Republic of Korea; ^6^ Department of Oral Anatomy, School of Dentistry, Chonbuk National University, Deokjin-Gu, Jeonju-Si, Jeollabuk-Do, Republic of Korea; ^7^ Institute of Oral Biosciences, Chonbuk National University, Deokjin-Gu, Jeonju-Si, Jeollabuk-Do, Republic of Korea

**Keywords:** amyloid β, age-related macular degeneration, transmission electron microscopy, retinal pigment epithelium, microarray, Gerotarget

## Abstract

Age-related macular degeneration (AMD) is a leading cause of blindness in the elderly. The two types of AMD are: dry and wet AMD. While laser-induced choroidal neovascularization has been used extensively in the studies of wet AMD, there is no established mouse model that fully recapitulates the cardinal features of dry AMD. A lack of appropriate mouse model for dry AMD has hampered the translational research on the pathogenesis of the disease and the development of therapeutic agents. We hypothesized that 5XFAD mice, an animal model for the study of Alzheimer’s disease, can be used as a mouse model for dry AMD with regard to the amyloid beta (Aβ) related pathology. In this study, the ultrastructure of the retinal pigment epithelium (RPE) of 5XFAD mice was analyzed using transmission electron microscopy. Of importance, the aged 5XFAD mice show ultrastructural changes in the RPE and Bruch’s membrane (BM) that are compatible with the cardinal features of human dry AMD, including a loss of apical microvilli and basal infolding of the RPE, increased BM thickness, basal laminar and linear deposits, and accumulation of lipofuscin granules and undigested photoreceptor outer segment-laden phagosomes. In microarray-based analysis, the RPE complex of the aged 5XFAD mice shows differential gene expression profiles consistent with dry AMD in the inflammation response, immune reaction pathway, and decreased retinol metabolism. Taken together, we suggest that aged 5XFAD mice can be used as a mouse model of dry AMD to study Aβ related pathology and develop a new therapeutic approaches.

## INTRODUCTION

Age-related macular degeneration (AMD) is a leading cause of blindness in the elderly [1]. The two types of AMD are: dry and wet AMD. In wet AMD, new blood vessels (known as choroidal neovascularization) grow into the macula and damage the retina. Dry AMD is characterized by the presence of drusen and atrophy of the retinal pigment epithelium (RPE) cells. While laser-induced choroidal neovascularization has been extensively used in the studies of wet AMD [2], there is no single mouse model that fully recapitulates the cardinal features of human dry AMD, mainly due to its multifactorial nature [3, 4]. Compared with humans, mice have a simpler Bruch's membrane (BM) structure without a macula. Furthermore, drusen, a hall-mark of dry AMD, is rarely found in mice. Therefore, it is essential to understand the ultrastructural pathology of dry AMD by analyzing basal deposits and BM at the ultrastructural level [4]. Despite the efforts to develop mouse models of dry AMD with various retinal lesions, a lack of an appropriate mouse model for dry AMD has impaired the translational research on the pathogenesis of the disease and the development of therapeutic agents. Thus, we aimed to validate the 5XFAD mouse, an animal model of Alzheimer's disease (AD) that harbors five familial AD mutations, as a novel dry AMD mouse model that clearly shows amyloid beta deposits in the RPE.

Amyloid beta (Aβ) has been implicated in the pathogenesis of both AD and AMD. Although 5XFAD mice are widely used as an animal model for the study of AD, the use of these mice for modeling dry AMD is largely unexplored. We recently suggested that 5XFAD mice could be used as a mouse model of dry AMD with regard to the Aβ-related pathology [5]. In line with AD, we hypothesized that dry AMD is a neurodegenerative disease that involves the accumulation of Aβ. For the evaluation of dry AMD, it is essential to validate the ultrastructural changes in the RPE. However, the ultrastructure of the RPE and BM of aged 5XFAD mice has not yet been investigated. Here, we hypothesized that the eyes of 5XFAD mice would show an ultrastructural pathology consistent with dry AMD as a result Aβ accumulation in the eye. In this study, we focused on the ultrastructure of the RPE and BM and conducted a gene analysis of 5XFAD mice to investigate dry AMD like pathology. Using transmission electron microscopy (TEM), we found that aged 5XFAD mice had ultrastructural changes in the RPE and BM that were compatible with the cardinal features of human dry AMD. Furthermore, we analyzed the differential gene expression profiles of the RPE complex in 5XFAD mice to link the anatomical changes to the microarray-based pathogenesis of dry AMD. Our results demonstrate that aged 5XFAD mice have the potential to be used as a novel dry AMD model.

## RESULTS

### Breakdown of tight junctions with Aβ accumulation in the RPE of the aged 5XFAD mice

**Figure 1:**
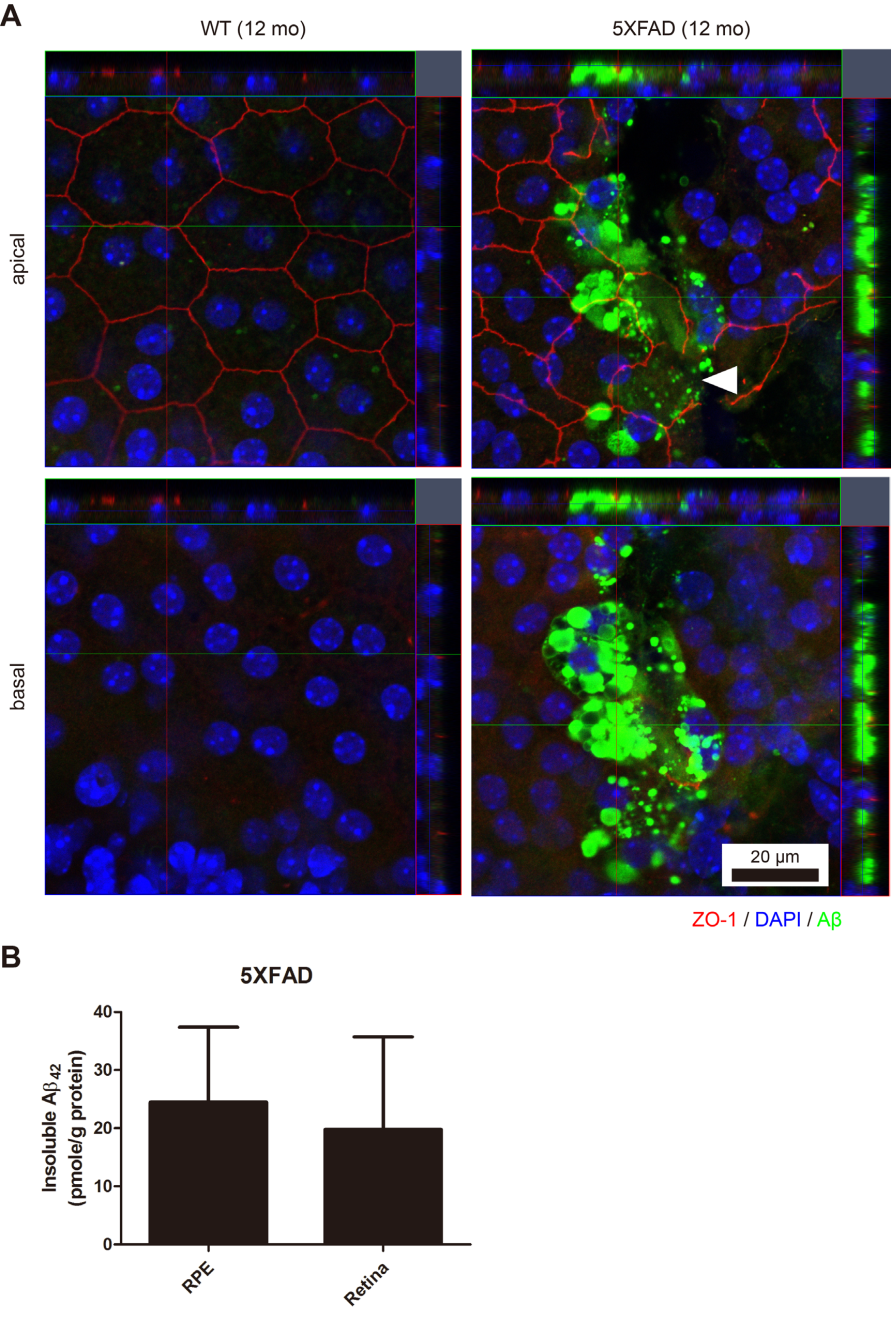
The RPE/choroid/scleral complex of aged 5XFAD mice with intracellular Aβ accumulation **A**. The RPE complex of the 12-month-old 5XFAD mice and the age-matched wild-type littermate mice (WT) were flat-mounted (*n* = 4). Representative images of 5XFAD mice show irregular apical tight junctions (ZO-1, red) compared to typical hexagonal tight junctions in the aged-matched WT mice. Arrowhead indicates disrupted tight junction of the RPE. Abundant accumulation of Aβ (green) were occasionally observed in the RPE complex of 5XFAD mice. Scale bar = 20 μm. **B**. Aβ_42_ levels of the RPE complex and the retina in 5XFAD mice (*n* = 7).

To validate the aged 5XFAD mouse as an animal model for dry AMD, we first investigated the integrity of the outer blood-retinal barrier. Compared with the hexagonal shape of the RPE in the 12 month-old wild type (WT) mice, the irregularly shaped apical tight junction (ZO-1) of the RPE in the 12 month-old 5XFAD mice indicated the breakdown of the outer blood-retinal barrier (Figure 1A). In addition to the loss of ZO-1 at the apical RPE, note that abundant Aβ deposits were present at the basal RPE juxta above the choroidal endothelial cells in the RPE/choroid complex flat-mounts (z-axis). These Aβ deposits at the RPE/choroid complex of the aged 5XFAD mice were also detected by ELISA as an insoluble form of Aβ_42_ (Figure 1B), not as a soluble form of Aβ_42._ The level of Aβ in the WT mice was below the detection limit of the ELISA.

### Increased thickness of Bruch's membrane (BM) in the aged 5XFAD mice

**Figure 2:**
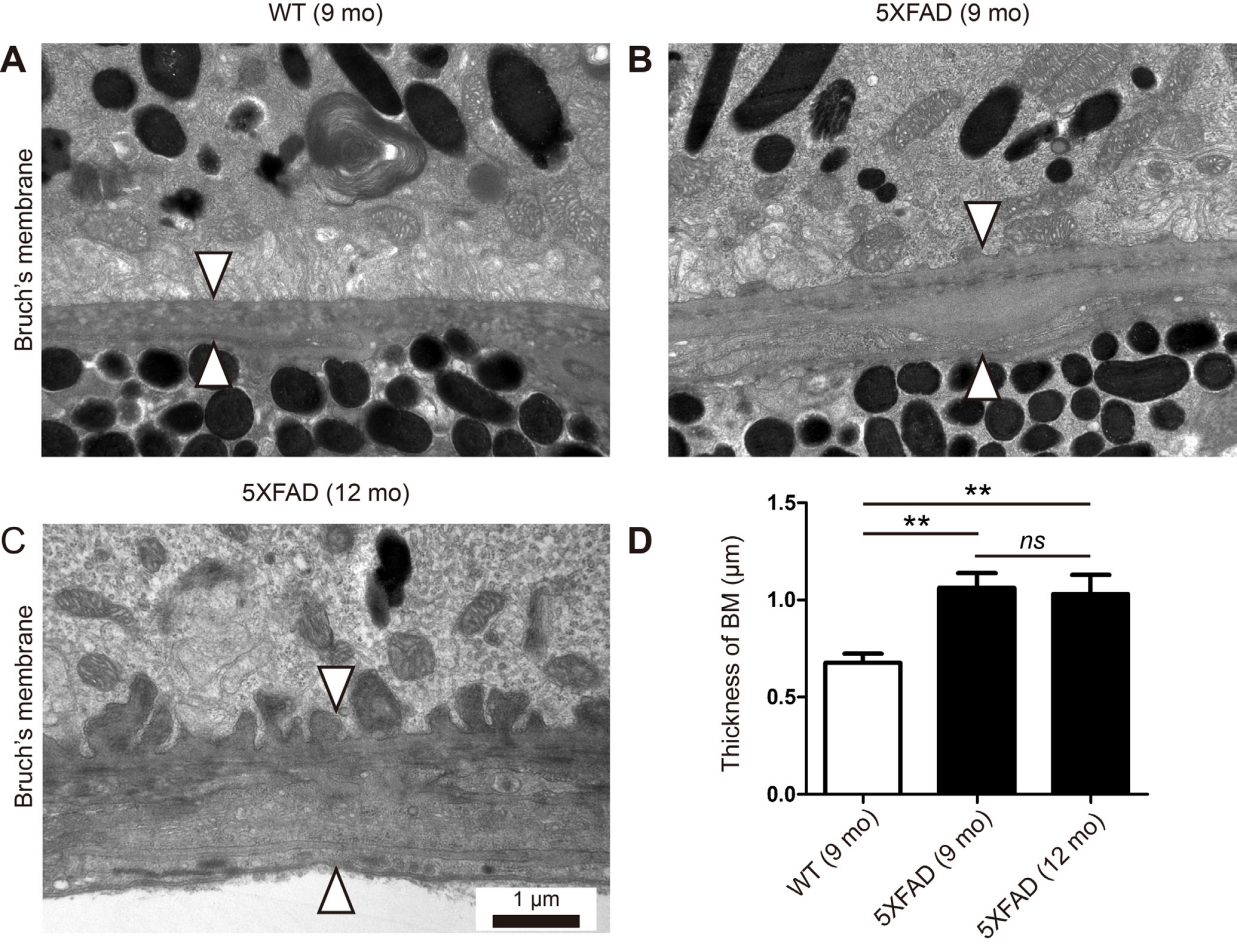
Thickening of Bruch's membrane in aged 5XFAD mice **A**. Representative transmission electron micrograph (TEM) images of the RPE complex from the 9 months old wild-type littermate mice (WT). **B**.-**C**. Representative TEM images of the RPE complex from the 9 and 12-month-old 5XFAD mice. (A) WT mice show normal structure and thickness of Bruch's membrane (arrowheads). (B-C) 5XFAD mice show thickening of Bruch's membrane (arrowheads) with basal linear deposits in outer collagenous layer (B) or with basal laminar deposits and loss of fenestration at choriocapillaris (C). Scale bars are 1 μm. **D**. Thickness of Bruch's membrane (BM). Error bars indicate s.e.m. (*n* = 8). One-way ANOVA and Tukey's post hoc tests, **P < 0.01.

Thickening of BM is one of the cardinal features of early dry AMD. We recently reported the thickening of BM in locations where Aβ deposits downregulate tight junctions in the RPE of aged 5XFAD mice [5]. However, this observation was based on H&E stained cross-sections, not on TEM-based ultrastructure. Thus, we analyzed the ultrastructure of the RPE/choroid of the aged 5XFAD mice in this study. The average thickness of BM was 0.68 ± 0.05 μm in the 9-month-old WT mice, 1.06 ± 0.08 μm in the 9-month-old 5XFAD mice, and 1.03 ± 0.10 μm in the 12-month-old 5XFAD mice, respectively (Figure 2A-2D). Although there was no significant change in the BM thickness between the 9-month-old and the 12-month-old 5XFAD mice, there was a significant increase in the BM thickness of the aged 5XFAD mice compared with that of the 9-month-old WT mice (*n* = 8, One-way ANOVA and Tukey's post hoc tests, *P* < 0.01, Figure 2D).

### Basal laminar and basal linear deposits at BM of 5XFAD mice

**Figure 3:**
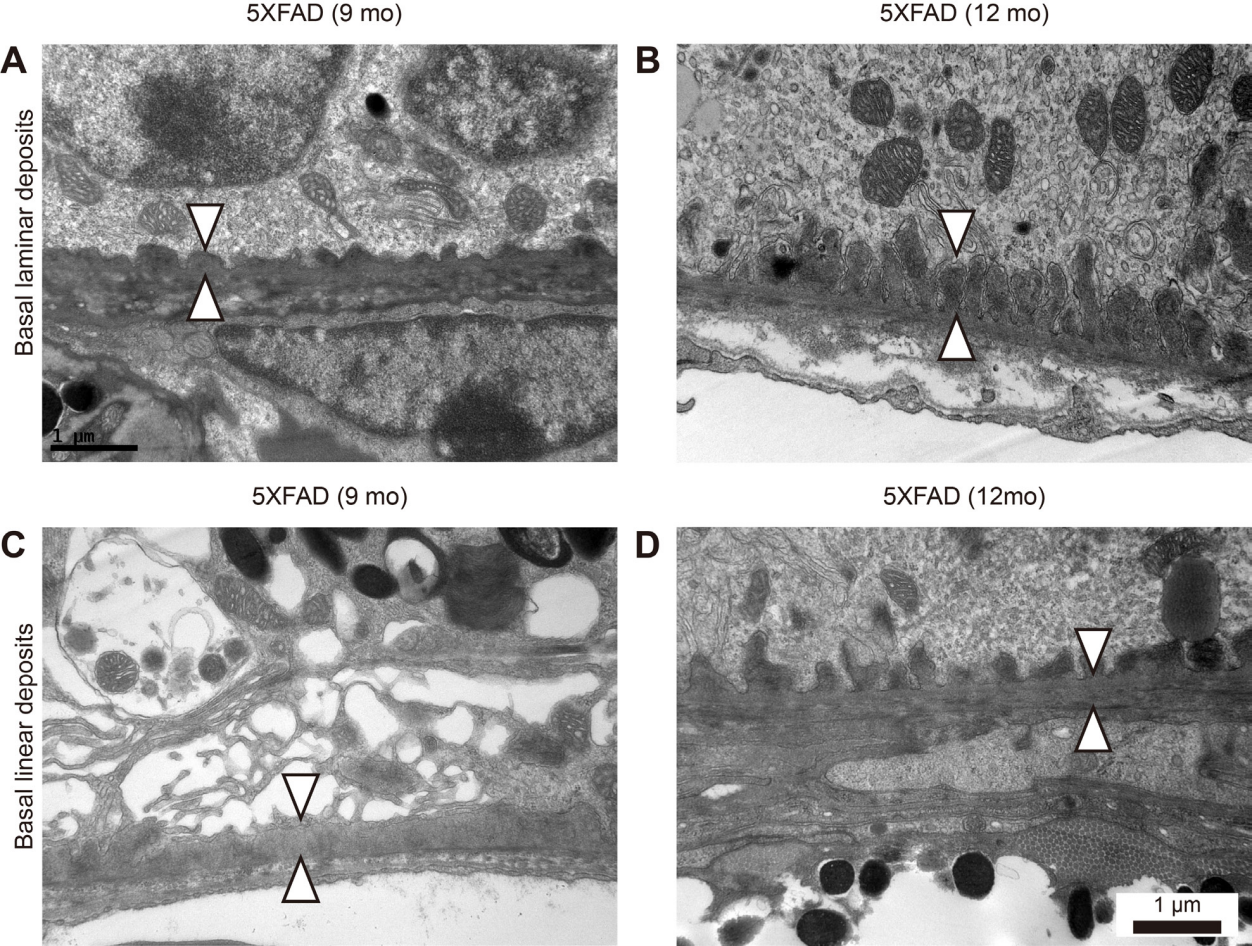
Basal laminar and linear deposits at Bruch's membrane in aged 5XFAD mice **A**.-**D**. Representative transmission electron micrograph (TEM) images of RPE complex from 9 and 12-month-old aged 5XFAD mice. (A) 5XFAD mice showed undulating surface of Bruch's membrane with basal laminar deposits (arrowheads). (B) 5XFAD mice showed elongated basal laminar deposits (arrowheads) with loss of basal infolding. (C) 5XFAD mice showed basal linear deposits (arrowheads) at inner collagenous layer of Bruch's membrane and cystic degeneration of RPE. (D) 5XFAD mice showed both basal laminar and basal linear deposits (arrowheads). Scale bars are 1 μm.

Drusen is a hallmark of dry AMD. Although we did not find large-drusen, we observed both basal laminar deposits and basal linear deposits (Figure 3). Basal laminar deposits were occasionally found in the 9-month-old WT mice. However, the deposit in the aged 5XFAD mice were continuous and long enough to form undulating basal laminar deposits (Figure 3A-3B). Basal linear deposits were found only in the aged 5XFAD mice (Figure 2B-2C and Figure 3C-3D) and they were associated with the increase in BM thickness. Note that the endothelial fenestrations were lost in the choriocapillaris underlying BM with the basal linear deposits (Figure 2C and Figure 3C).

### Loss of apical microvilli and basal infolding in the RPE of the aged 5XFAD mice

**Figure 4:**
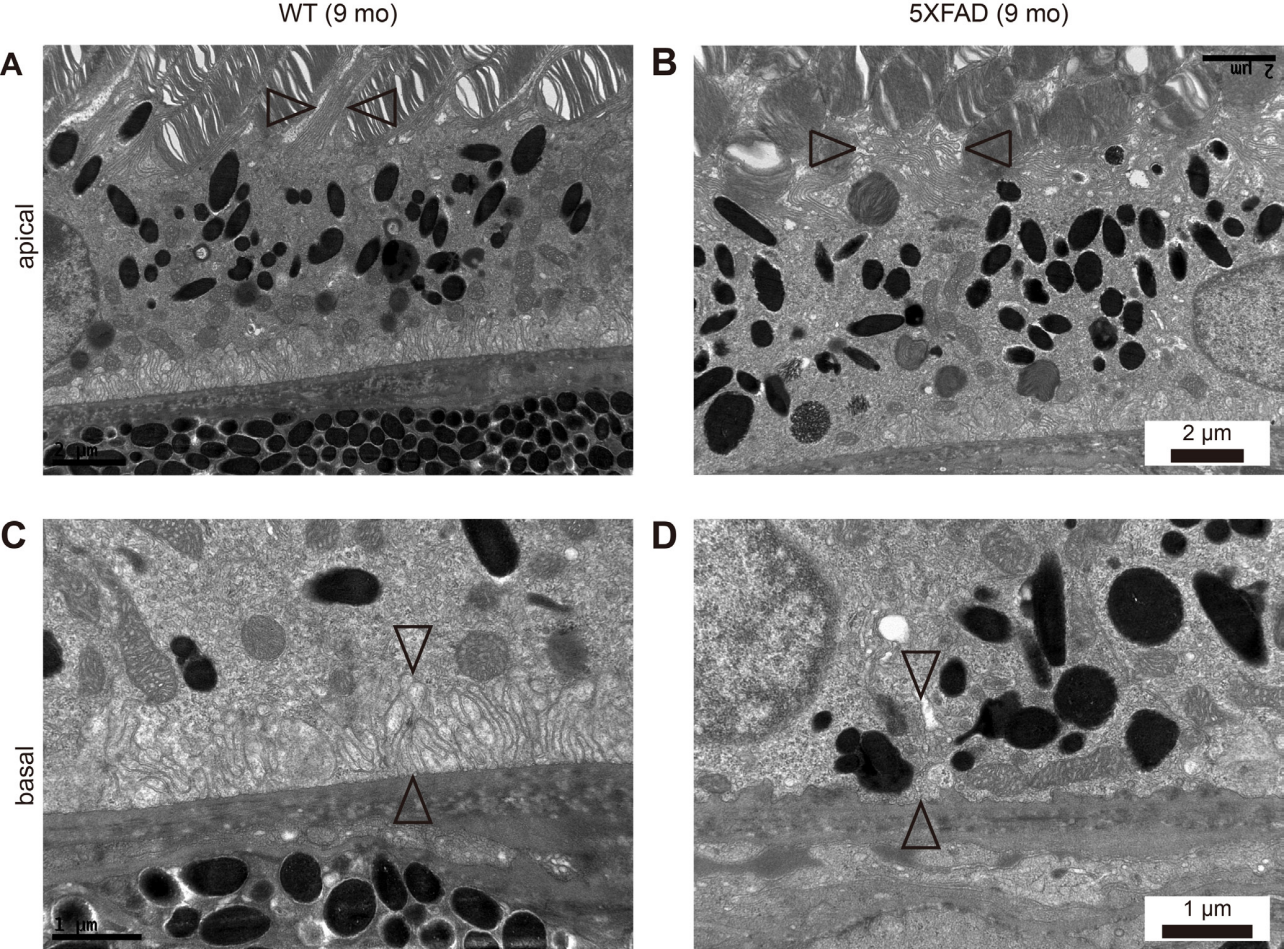
Loss of apical microvilli and basal infolding in aged 5XFAD mice **A**., **C**. Representative transmission electron micrograph (TEM) images of RPE complex from 9-month-old wild-type littermate mice (WT). **B**., **D**. Representative TEM images of RPE complex from 9-month-old 5XFAD mice. (A) WT mice showed normal microvilli (arrowheads) surrounding photoreceptor outer segments (POS) at apical RPE. (B) 5XFAD mice showed disorganized apical microvilli (arrowheads) and POS. Scale bars are 2 μm. (C) WT mice showed well organized basal infolding (arrowheads) above Bruch's membrane, supporting intracellular organelles at basal RPE. (D) 5XFAD mice showed loss of basal infolding (arrowheads) and unsupported intracellular organelles juxta above Bruch's membrane. Scale bars are 1 μm.

The phagocytosis of shed photoreceptor rod outer segments (POS) by the RPE is essential for the maintenance of retinal function. In the 5XFAD mice, we observed a loss of the apical microvilli (Figure 4A-4B). We performed a microarray-based differential expression gene analysis to investigate the relationship between the RPE structure and gene expression. Note the downregulation of *Itgav* (0.591 fold, *P* = 0.0293); the RPE requires αvβ5 integrin for binding to the shed POS [6]. *Cldn1* (Claudin 1, 0.644 fold, *P* = 0.0266) is localized to the cilia of the RPE [7] and *Rrh* (retinal pigment epithelium derived rhodopsin homolog, 0.641 fold, *P* = 0.00466), also known as peropsin, is localized to the microvilli in the RPE [8]. Thus, decreased expression levels of these genes might be strongly associated with a loss of microvilli (Figure 4). In addition, *F3* (coagulation factor III, 0.613 fold, *P* = 0.00098) is known to be upregulated in the RPE after POS uptake [9], as well as in the macrophages after phagocytosis [10]. Thus, downregulation of F3 might be related to the decrease in POS uptake in the RPE resulting from the loss of microvilli. The differentially expressed gene profile associated with the apical microvilli is shown in Supplementary Table S1.

We also observed a loss of basal infolding in the aged 5XFAD mice (Figure 4C-4D), which supports intracellular organelles although the precise role of basal infolding is not well established. A loss of basal infolding, which led to direct contact between the intracellular vesicles, including lipofuscin and phagosomes, and BM, thereby affected the thickness of BM.

### Increased number of intracellular granules of the RPE in the aged 5XFAD mice

**Figure 5:**
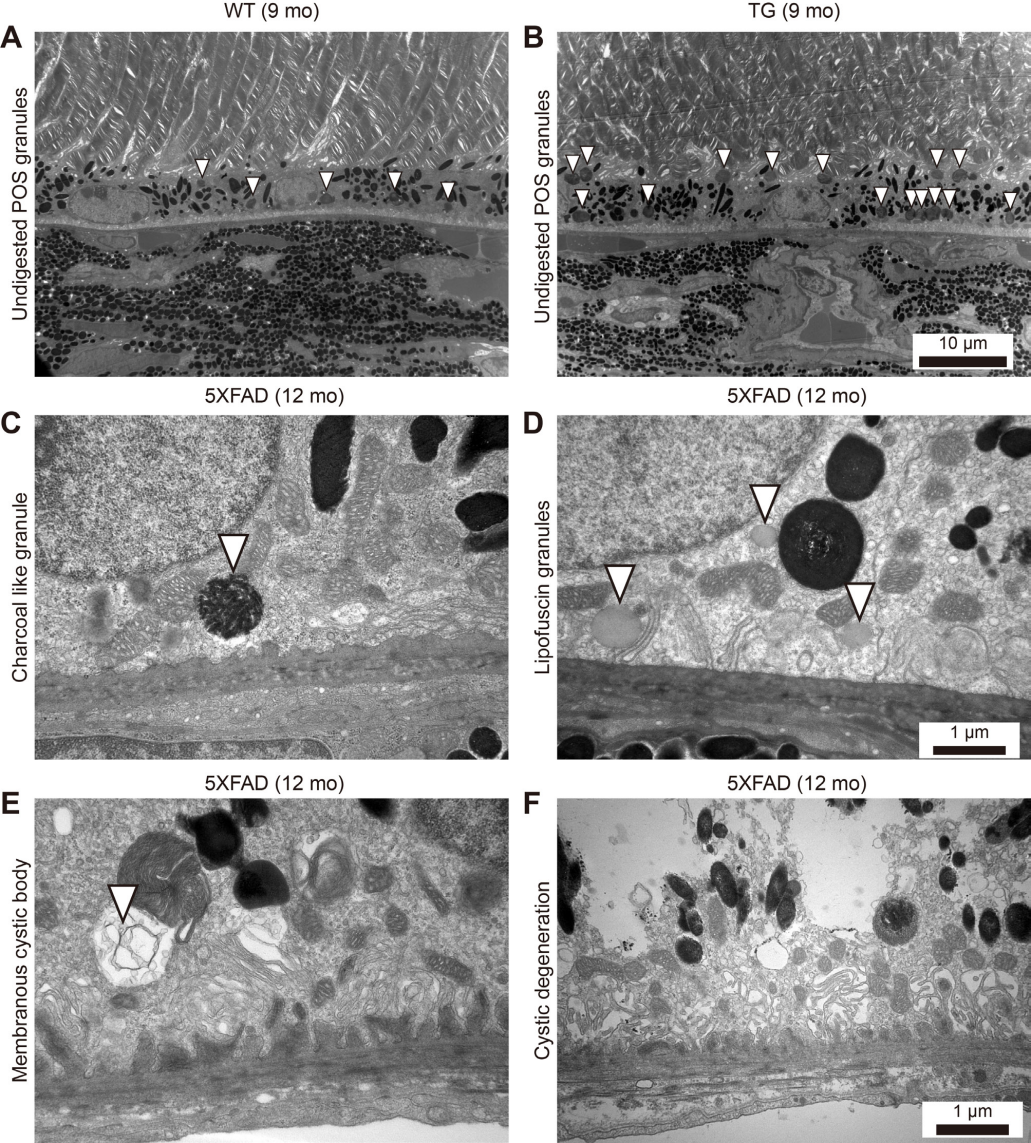
Increased number of intracellular granules of the RPE in aged 5XFAD mice **A**.-**D**. Representative transmission electron micrograph (TEM) images of the RPE complex from 9 and 12-month-old 5XFAD mice and 9-month-old wild-type littermate mice. (A) WT mice showed normal distribution of melanin pigments and lysosomal granules (arrowheads). (B) 5XFAD mice showed increased number of undigested photoreceptor cell outer segments (POS) in lysosomal granules (arrowheads). Scale bars are 10 μm. (C) 5XFAD mice occasionally showed a charcoal-like granules (arrowhead) with loss of basal infolding. (D) 5XFAD mice showed increased number of lipofuscin granules with disrupted basal infolding. Scale bars are 1 μm. **E**. 5XFAD mice showed membranous cystic body (arrowhead). **F**. 5XFAD mice showed marked cystic degeneration with ruptured cystic vacuole and disseminated undigested particles in RPE. Scale bars are 1 μm.

One of the important functions of the RPE is to phagocytose POS and digest them for recycling. However, the incomplete digestion of POS may lead to the accumulation of undigested POS granules in phagosomes or accumulation of lipofuscin granules in the RPE. Indeed, the 9-month-old 5XFAD mice RPE contained more undigested POS granules (Figure 5B, 10.4 ± 0.8 per high power field, *n* = 8) than the age-matched WT mice (Figure 5A, 5.0 ± 1.1 per high power field, *n* = 8, *P* = 0.0017). In addition to undigested POS-laden phagosomes, charcoal like granules (Figure 5C) or increased numbers of lipofuscin granules (Figure 5D) were observed in the RPE of the 5XFAD mice, especially at the basal side of the RPE, where the basal infolding was lost.

### Differentially expressed gene profile associated with retinol metabolism and the inflammatory pathway in the RPE complex of the aged 5XFAD mice

**Figure 6:**
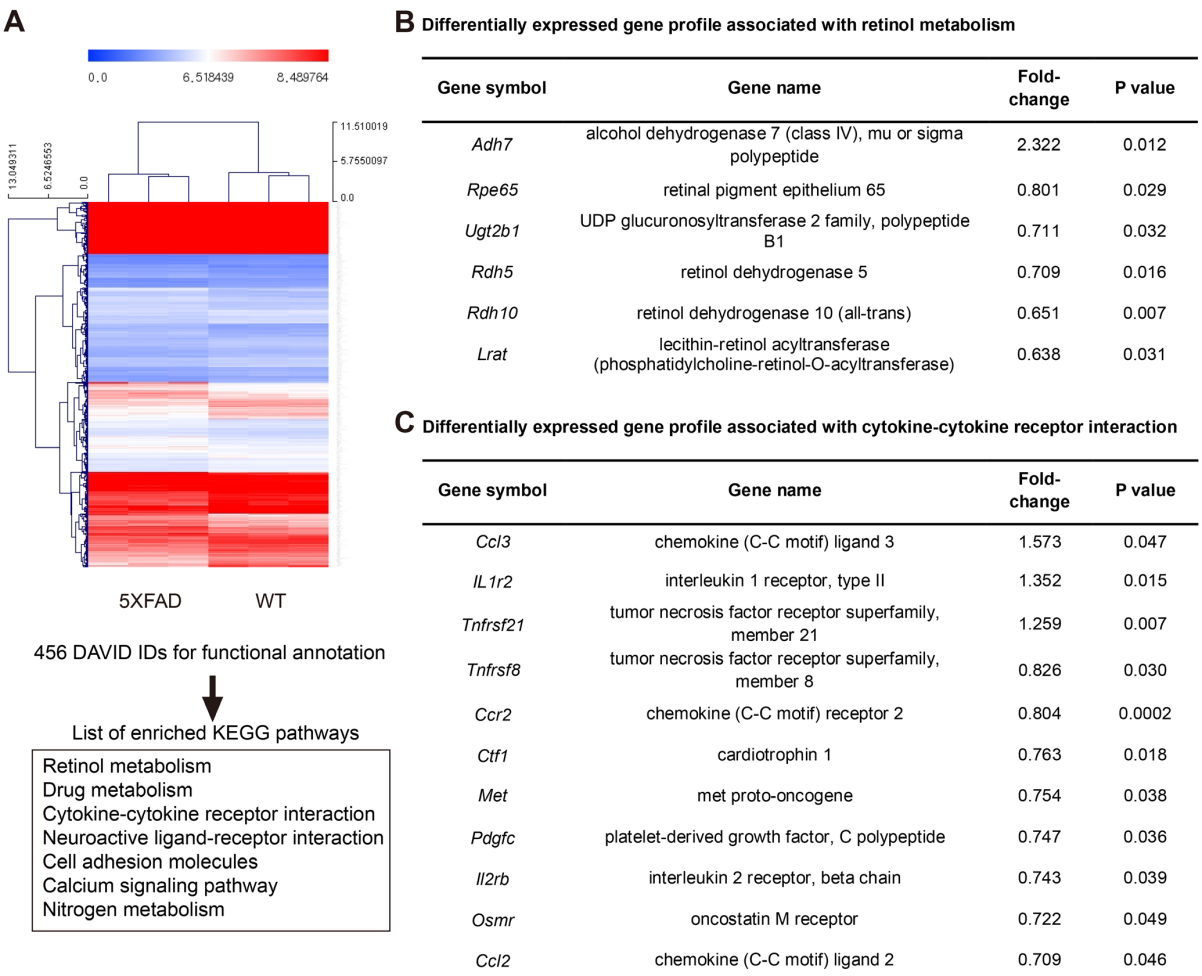
Microarray based differentially expressed gene profile with retinol metabolism and cytokine-cytokine receptor interaction in the RPE complex of 5XFAD mice **A**. Hierarchical clustering map of 5XFAD mice and littermate wild type mice (WT) using gene identifiers (IDs) with 1.2 fold change with p value < 0.05, leading to list of enriched KEGG pathways in DAVID analysis for functional annotation. **B**. Differentially expressed gene profile associated with retinol metabolism in 5XFAD mice. **C**. Differentially expressed gene profile associated with cytokine-cytokine receptor interaction in 5XFAD mice.

In the aged 5XFAD mice, we observed an intracellular membranous cystic body in the RPE as well as cystic degeneration (Figure 5E-5F). Evidently, the cystic degeneration of the RPE led to RPE dysfunction other than phagocytosis or vice versa. One of the important roles of the RPE is retinol metabolism. Our microarray-based differential expression gene analysis revealed the down-regulation of several key enzymes involved in retinol metabolism (Figure 6A-6B). *Adh7* (2.322 fold, *P* = 0.012) is associated with retinol metabolism. Furthermore, *Rpe65* (0.801 fold, *P* = 0.0290) is a major player in retinol metabolism, and its loss has been directly linked to retinal degeneration. Additional enzymes involved in retinol metabolism, including *Rdh5* (0.709 fold, *P* = 0.0159), *Rdh10* (0.651 fold, *P* = 0.0070), *Lrat* (0.638 fold, *P* = 0.031473), and *Ugt2b1* (0.711, *P* = 0.032628) were also decreased in the aged 5XFAD mice, which suggests overall dysfunctional retinol metabolism in the RPE (Figure 6B). In addition to the genes related to retinol metabolism, *Ttr* (transthyretin, 0.848 fold, *P* = 0.0033) acts as a carrier of retinol (vitamin A) through its association with retinol-binding protein (RBP) in the blood and CSF. *Rbp1* (retinol binding protein 1, 0.812 fold, *P* = 0.0247) was also down regulated in the aged 5XFAD mice. In addition, *Trf* (transferrin, 0.717 fold, *P* = 0.0143) is transcriptionally regulated by retinoic acid [11].

It has been suggested that the inflammatory process is closely associated with the pathogenesis of dry AMD. Several mouse models using the inflammatory pathway have been developed. The aged 5XFAD mice show differential gene expression in cytokine-cytokine receptor interaction, and some of these interactions are closely related to the inflammatory response or immune reaction (Figure 6C). In the aged 5XFAD mice, the increased *Ccl3* (1.57 fold, *P* = 0.047), which interacts with CCL4 to attract macrophages, monocytes, and neutrophils, and *Mpeg1* (1.247 fold, *P* = 0.0390) suggested the possible involvement of macrophages in the RPE complex. Interestingly, the decreased *Ccl2* (0.71 fold, *P* = 0.046) and *Ccr2* (0.804 fold, *P* < 0.0001) in the aged 5XFAD mice is reminiscent of the *Ccl2* or *Ccr2* deficient mouse model of dry AMD (Figure 6C) [12]. Additionally, a mutation in *Cdhr1* (0.549 fold, *P* = 0.0089) has a known association with autosomal recessive retinal dystrophy [13].

Other differentially expressed gene profiles in the RPE complex of the aged 5XFAD mice are listed in Supplementary Table S2. *Ilr1r2* (1.352 fold, *P* = 0.0271) may function as an IL-1 decoy by interacting with the IL-1 receptor accessory protein [14]. *Gsto1* (3.80 fold, *P* = 0.010) and *Mgst1* (1.344 fold, *P* = 0.0276) are related to glutathione (GSH) homeostasis, and their upregulation leads to a decrease in reduced glutathione [15]. *Sema3c* (0.537 fold, *P* = 0.0358) is a known inhibitor of pathological angiogenesis [16]; therefore, downregulation of *Sema3c* may indicate the potential development of pathological angiogenesis in the 5XFAD mice.

## DISCUSSION

Aβ_42_ has been implicated in dry AMD based on the parallel findings in AD and AMD [17]. We recently suggested that the 5XFAD mouse could be a potential mouse model of dry AMD with regard to the Aβ-related pathology [5]. In one mouse of dry AMD, *APOE4* mice maintained on a high fat diet, Aβ accumulates in sub-RPE deposits [18]. These mice also exhibit ZO-1 tight junction breakdown in RPE flat-mounts and thickening of BM. In this study, we demonstrated that the aged 5XFAD mice show outer blood-retinal barrier breakdown with Aβ accumulation. In addition, the aged 5XFAD mice show ultrastructural changes in the RPE and BM that are compatible with the cardinal features of human dry AMD, including a loss of apical microvilli and basal infolding of the RPE, increased BM thickness, basal laminar and linear deposits, and accumulation of lipofuscin granules and undigested POS-laden phagosomes.

We noted that these pathognomonic ultrastructural changes in the RPE of the aged 5XFAD mice have also been found in previously established AMD animal models with various underlying mechanisms (Supplementary Table S3). Ramkumar et al. [4] have summarized the genetics and retinal pathology of other dry AMD models. The basal laminar and linear deposits beneath the RPE layer with a loss of basal infolding and numerous cystic vacuoles in the 5XFAD mice have also been found in *neprilysin* gene-disrupted mice [19], another mouse model that suggests a possible role for Aβ in AMD. In mcd/mcd mice, a transgenic mouse line (mcd/mcd) expressing a mutated form of cathepsin D that is enzymatically inactive and thus impairs the processing of phagocytosed POS in the RPE cells, TEM has revealed the presence of basal laminar and linear deposits, which are considered to be the hallmarks of AMD, similar to those observed in the 5XFAD mice [20]. Marked vacuolization and increases in lipofuscin granules and intracellular dense bodies have been observed in *Ccl2*-/- mice with TEM [12] and are also present in the aged 5XFAD mice. *Cfh+/-* mice fed with a high fat, cholesterol-enriched diet have RPE and photoreceptor dysfunction in response to basal laminar deposits [21].

The complement factor H (CFH) polymorphism (Y402H) is a risk factor for AMD [22, 23]. The studies of multiple mouse models of AMD including human *APOE4* knock-in mice [18], human *CFH* transgenic mice [24], and *Cfh*+/- mice [21] have focused on the complement activation. The advantage of using these *CFH* related mouse models is the support for a role for complement activation in AMD pathogenesis comes from studies implicating variations in the *CFH* gene as the strongest genetic factor associated with risk for AMD [22, 23]. The precise mechanisms of complement system dysregulation in AMD are unknown, although there are several candidate molecules including Aβ. Aβ, a constituent of drusen, is a known activator of the complement system. Aβ is associated with drusen in human AMD eyes, but not with drusen in normal eyes [25]. In particular, oligomeric Aβ presents in drusen from human AMD eyes [26]. In addition, Aβ accumulates and co-localizes with activated complement components within drusen [27, 28]. Although we did not investigate the molecular mechanism of Aβ contribution to the pathogenesis of dry AMD, we directly used Aβ overexpressing mouse to recapitulate dry AMD-like pathology. These dry AMD-like pathology in 5XFAD mice might be associated with complement system dysregulation by Aβ. In this regard, 5XFAD mice have an advantage to study the contribution of Aβ to the complement system dysregulation without genetic modification of *CFH*-related genes.

Furthermore, the RPE complex of the aged 5XFAD mice shows differential gene expression profiles consistent with dry AMD in the inflammation response, immune reaction pathway, and decreased retinol metabolism. However, ultrastructural and gene profile changes have not been studied together in dry AMD animal models. Intriguingly, we can link the 5XFAD mouse differential gene expression profile with key ultrastructural changes, such as the loss of microvilli. Loss of microvilli and basal infolding has been found in various dry AMD models [4]. However, we, for the first time, suggest that these changes might be linked to the decreased expression of *Itgav, Cldn1,* and *Rrh*. Note that RPE phagocytosis shows a remarkable specificity for POS mediated by αvβ5 integrin [6, 29]. Most importantly, the decreased *Ccl2* and *Ccr2* in the aged 5XFAD mice is reminiscent of the *Ccl2* or *Ccr2* deficient mouse model of AMD [12], because it has been suggested that the inflammatory process is closely related to the pathogenesis of dry AMD. In addition, we postulate that the downregulation of the genes related to retinol metabolism is the result of Aβ induced RPE dysfunction. Taken together, our findings suggest that aged 5XFAD mice can be used as a dry AMD mouse model.

A limitation of this study is the lack of extensive geographic atrophy in the aged 5XFAD mice, another cardinal feature of end stage dry AMD. Considering that no other animal models of dry AMD have completely recapitulated geographic atrophy, it is not surprising that we did not find geographic atrophy in the 12-month-old 5XFAD mice. Nonetheless, for the first time, this study demonstrated that 5XFAD mice show ultrastructural changes consistent with those of dry AMD.

Regarding the Aβ_42_ related pathology in AD and AMD [17], we first suggested that 5XFAD mice could be used as a mouse model of dry AMD [5]. Recently, hyperspectral imaging has detected amyloidopathy in the retina before the onset of cognitive dysfunction in 5XFAD mice [30]. We thought that these findings indicated that 5XFAD mice could be used not only for the study of AMD, but also for the study of an early surrogate marker for the development of AD. Thus, we can use the eye as a window to the brain in the clinic.

In conclusion, we demonstrated that the ultrastructural changes in aged 5XFAD mice are comparable with the cardinal features of dry AMD. Thus, we suggest that aged 5XFAD mice can be used as a mouse model of dry AMD to study Aβ-related pathology and develop new therapeutic approaches.

## MATERIALS AND METHODS

### Animals

This study used 5XFAD mice that were purchased from the Jackson Laboratory (Bar Harbor, ME, USA). Mice (5XFAD) overexpress mutant human amyloid precursor protein 695 with the Swedish mutation (K670N, M671L: elevate the production of total Ab), Florida mutation (I716V: elevates the production of Ab42 specifically), and London mutation (V717I: elevates the production of Ab42 specifically) and human presenilin 1 with 2 FAD mutations (M146L and L286V: elevate the production Ab42 specifically)[5, 31]. All 5XFAD transgenic mice (Tg6799, B6 ⁄ SJL hybrid background) used were heterozygotes with respect to the transgene, and nontransgenic wild-type littermate (WT) mice served as controls. In addition, to eliminate any possible confounding factor from carrying rd1/rd1 genotype [32], all 5XFAD mice used in this study were confirmed not to carry rd1 gene (WT/WT) (Supplementary Figure S1). Mice were maintained under a 12 h dark-light cycle. All animal experiments in this study were in strict agreement with the Association for Research in Vision and Ophthalmology Statement for the Use of Animals in Ophthalmic and Vision Research and the guidelines of the Gwangju Institute of Science and Technology Animal Care and Use Committee.

### Immunofluorescence staining

We performed immunofluorescence staining of RPE complex as previously described [33]. After deep anesthesia, mice were sacrificed and enucleated eyes were fixed in 4% paraformaldehyde for 24 h. RPE complex was incubated in Perm/Block solution (0.2% Triton-X 100 and 0.3% BSA in PBS) at RT for 1 h. Then, it was incubated overnight at 4°C with primary antibodies against rabbit anti-ZO-1 (1:100, cat# 61-7300, Invitrogen, Carlsbad, CA, USA) and mouse anti-Aβ (1:100, 4G8, Covance, Princeton, NJ, USA). After washing with PBS, it was incubated at RT for 2 h with secondary antibodies (Alexa Fluor 488 donkey anti-mouse IgG, 1:200 and Alexa Fluor 594 donkey anti-rabbit Ig G, 1:200). After washing with PBS, it was counterstained with 10 mg/ml DAPI (Sigma Aldrich, St. Louis, MO, USA). After washing with PBS, the RPE/choroid complex was mounted with Fluoromount™ Aqueous Mounting Medium (Sigma Aldrich) and observed under confocal microscope (LSM710, Carl Zeiss, Oberkochen, Germany).

### Aβ42 ELISA

For Aβ_42_ ELISA, enucleated eyes were immediately separated into neural retina and RPE/choroid complex. Then, each sample was snap-frozen in liquid nitrogen and kept in -80°C until analysis. We measured Aβ level in retina and RPE complex with ELISA Human Aβ_42_ Ultrasensitive ELISA Kit (Invitrogen, cat#KHB3544) according to the manufacturer's instructions. Each sample was lysed with RIPA buffer and centrifuged at 13,000 RPM for 20 min. Supernatants were designated to RIPA soluble fraction of Aβ_42_. Then, the pellet, RIPA insoluble fraction, was further lysed with 70% formic acid and sonicated briefly. Then, the sample was ultracentrifuged at 100,000 g for 1 hr. The supernatants after ultracentrifugation were isolated and neutralized by adding 1M Tris (pH 11). This fraction was designated as RIPA insoluble sample. Total tissue protein was measured from each sample using BCA kit.

### Transmission electron microscopy (TEM)

For TEM, 8 eyes from 4 mice were used from each group of 9-month-old male WT, 9-month-old male 5XFAD, and 12-month-old male 5XFAD. Enucleated eyes were immediately fixed in 1.5% paraformaldehyde/1.5% glutaraldehyde in 0.1 M sodium cacodylate buffer (pH 7.4), postfixed in 1% osmium tetroxide in 0.1 M sodium cacodylate buffer (pH7.4), dehydrated in a graded ethanol series, and embedded in epoxy resin according to standard protocols. Semithin sections (1-μm thick) were stained with toluidine blue; ultrathin sections were stained with uranyl acetate and lead citrate and were examined with an electron microscope (H-7650, HITACHI, Japan). The thickness of Bruch's membrane was measured in two representative images per each case (*n* = 8). The thinnest and thickest parts of BM were measured and the average thickness was determined [34].

### RNA extraction and microarray based gene expression profiling

In the present study, we performed global gene expression analyses using Affymetrix GeneChip^®^ Mouse Gene 2.0 ST oligonucleotide arrays. For microarray-based expression profiling, RPE/choroid complex was isolated from neural retina immediately after enucleation. Then, each sample was snap-frozen in liquid nitrogen and kept in -80 °C. Total RNA was isolated from RPE/choroid complex using TRIzol reagent (Invitrogen) according to the manufacturer's instructions. RNA quality was assessed by Agilent 2100 bioanalyser (Agilent Technologies, USA), and quantity was determined by ND-1000 spectrophotometer (NanoDrop Technologies, USA). Per RNA sample, 300 ng were used as input into the Affymetrix procedure according to the manufacturer's recommended protocol (http://www.affymetrix.com). For data analysis, Affymetrix GeneChip^®^ Human Gene 2.0 ST oligonucleotide array was scanned using Affymetrix Model 3000 G7 scanner and the image data was extracted through Affymetrix Commnad Console software1.1. The raw.cel file generated through above procedure meant expression intensity data and was used for the next step. Expression data were generated by Affymetrix Expression Console software version1.1. For the normalization, RMA (Robust Multi-Average) algorithm implemented in Affymetrix Expression Console software was used. In order to classify the co-expression gene group which has similar expression pattern, we performed Hierarchical clustering in MEV (MultiExperiment Viewer) software 4.4 (www.tm4.org) using differentially regulated transcripts with a fold change (FC) greater than 1.2. The web-based tool, DAVID (the Database for Annotation, Visualization, and Integrated Discovery) was used to perform the biological interpretation of differentially expressed genes [35]. Then, these genes were classified based on the information of gene function in Gene ontology, KEGG Pathway database. (http://david.abcc.ncifcrf.gov/home.jsp). Array data are available in the GEO database under GSE85408. Database from microarray were available via files in online Supporting Information.

### Statistical analysis

Statistical analyses were performed using SPSS software version 18.0 (SPSS Inc., Chicago, IL, USA). Two-tailed unpaired T-test or one-way ANOVA with Tukey post-hoc tests were used. *P* values less than 0.05 were considered to be statistically significant. Data and figures are depicted as mean ± s.e.m.

### Data availability

Array data are available in the GEO database under GSE85408. Database from microarray were available via files in online Supporting Information.

## SUPPLEMENTARY MATERIALS FIGURES AND TABLES




