# Supplementary material for: Dry age-related macular degeneration like pathology in aged 5XFAD mice: Ultrastructure and microarray analysis
Source: Oncotarget. 2017 Apr 8;8(25):40006–18. doi: 10.18632/oncotarget.16967 (PMC5522269; doi:10.18632/oncotarget.16967)
Supplement: Supplementary file 1 [file oncotarget-08-40006-s001.pdf]

Dry age-related macular degeneration like pathology in aged 5XFAD mice: Ultrastructure and microarray analysis

Supplementary Material

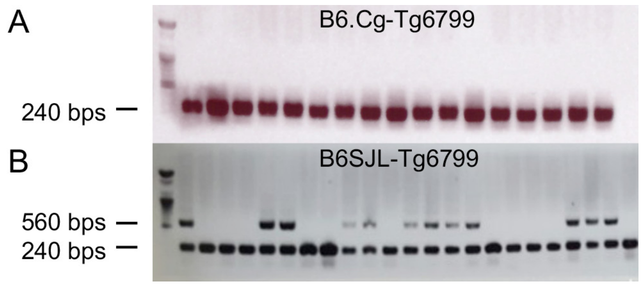

Supplementary Figure S1. The representative results of genotyping of *Pde6b^{rd1}* in 5XFAD mice with (A) B6 background and (B) SJL background. Bands at 560 bps indicate *Pde6b^{rd1}* allele. We only used 5XFAD mice that does not carry the retinal degeneration allele *Pde6b^{rd1}* (WT/WT: single band at 240 bps).

For Supplementary Tables 1,2,3 see in supplementary Files
