# Supplementary material for: Dry age-related macular degeneration like pathology in aged 5XFAD mice: Ultrastructure and microarray analysis
Source: Oncotarget. 2017 Apr 8;8(25):40006–18. doi: 10.18632/oncotarget.16967 (PMC5522269; doi:10.18632/oncotarget.16967)
Supplement: Supplementary file 2 [file oncotarget-08-40006-s002.doc]

| **Supplementary Table S1. Selected genes associated with phagocytosis and microvilli in differential gene expression profile of 5XFAD mice.** | | | |
| --- | --- | --- | --- |
| Gene symbol | Gene name | Fold change (TG/WT) | p-value |
| *Itgav* | integrin alpha V | 0.591 | 0.029 |
| *Cldn1* | claudin 1 | 0.644 | 0.026 |
| *Rrh* | retinal pigment epithelium derived rhodopsin homolog | 0.641 | 0.004 |
| *F3* | coagulation factor III | 0.613 | 0.009 |

| **Supplementary Table S2. Differentially expressed gene profile in RPE complex of 5XFAD mice.** | | | | |
| --- | --- | --- | --- | --- |
| Probe Set ID | Gene symbol | Gene name | Fold change (TG/WT) | p-value |
| 17322075 | *Krt4* | keratin 4 | 9.459 | 0.032 |
| 17321966 | *Krt5* | keratin 5 | 3.882 | 0.032 |
| 17360216 | *Gsto1* | glutathione S-transferase omega 1 | 3.800 | 0.010 |
| 17269347 | *Krt13* | keratin 13 | 2.443 | 0.006 |
| 17403058 | *Adh7* | alcohol dehydrogenase 7 (class IV), mu or sigma polypeptide | 2.322 | 0.012 |
| 17467471 | *Igkv4-50* | immunoglobulin kappa variable 4-50 | 2.270 | 0.035 |
| 17284548 | *Ighv1-34* | immunoglobulin heavy variable 1-34 | 2.232 | 0.039 |
| 17467449 | *Igkv4-62* | immunoglobulin kappa variable 4-62 | 2.101 | 0.049 |
| 17449262 | *Tmprss11g* | transmembrane protease, serine 11g | 2.072 | 0.039 |
| 17357640 | *Ms4a4a* | membrane-spanning 4-domains, subfamily A, member 4A | 2.015 | 0.013 |
| 17449251 | *Tmprss11a* | transmembrane protease, serine 11a | 1.949 | 0.007 |
| 17423030 | *Penk* | preproenkephalin | 1.935 | 0.049 |
| 17460465 | *Asprv1* | aspartic peptidase, retroviral-like 1 | 1.884 | 0.015 |
| 17352913 | *Dsc3* | desmocollin 3 | 1.880 | 0.024 |
| 17366928 | *Mir669o* | microRNA 669o | 1.869 | 0.029 |
| 17302878 | *Zic2* | zinc finger protein of the cerebellum 2 | 1.790 | 0.014 |
| 17273304 | *Cbr2* | carbonyl reductase 2 | 1.686 | 0.008 |
| 17546745 |  | --- | 1.654 | 0.027 |
| 17239817 | *Enpp3* | ectonucleotide pyrophosphatase/phosphodiesterase 3 | 1.641 | 0.019 |
| 17517097 | *Plet1* | placenta expressed transcript 1 | 1.575 | 0.017 |
| 17266967 | *Ccl3* | chemokine (C-C motif) ligand 3 | 1.573 | 0.047 |
| 17514910 | *Olfr830* | olfactory receptor 830 | 1.554 | 0.018 |
| 17289602 | *Adamts6* | a disintegrin-like and metallopeptidase (reprolysin type) with thrombospondin type 1 motif, 6 | 1.527 | 0.032 |
| 17540743 | *Slx* | Sycp3 like X-linked | 1.520 | 0.030 |
| 17499818 | *Defb2* | defensin beta 2 | 1.511 | 0.048 |
| 17244217 | *Rmst* | rhabdomyosarcoma 2 associated transcript (non-coding RNA) | 1.508 | 0.036 |
| 17274346 | *5730507C01Rik* | RIKEN cDNA 5730507C01 gene | 1.481 | 0.027 |
| 17466743 | *Npvf* | neuropeptide VF precursor | 1.479 | 0.006 |
| 17401999 | *Mir669n* | microRNA 669n | 1.477 | 0.014 |
| 17229533 | *Gm7694* | predicted gene 7694 | 1.475 | 0.004 |
| 17449288 | *Tmprss11b* | transmembrane protease, serine 11B | 1.471 | 0.044 |
| 17392828 | *LOC102633071* | uncharacterized LOC102633071 | 1.465 | 0.038 |
| 17491741 |  | --- | 1.460 | 0.018 |
| 17381879 | *St8sia6* | ST8 alpha-N-acetyl-neuraminide alpha-2,8-sialyltransferase 6 | 1.459 | 0.048 |
| 17308099 | *Adam28* | a disintegrin and metallopeptidase domain 28 | 1.458 | 0.034 |
| 17311512 | *Mal2* | mal, T cell differentiation protein 2 | 1.456 | 0.005 |
| 17337152 |  | --- | 1.456 | 0.050 |
| 17298775 | *Anxa8* | annexin A8 | 1.453 | 0.021 |
| 17281748 | *Trim9* | tripartite motif-containing 9 | 1.453 | 0.033 |
| 17506095 | *Necab2* | N-terminal EF-hand calcium binding protein 2 | 1.453 | 0.041 |
| 17459294 | *Igkv1-133* | immunoglobulin kappa variable 1-133 | 1.453 | 0.049 |
| 17334666 | *Tpsb2* | tryptase beta 2 | 1.450 | 0.023 |
| 17438189 | *Rasl11b* | RAS-like, family 11, member B | 1.449 | 0.031 |
| 17309041 | *Dach1* | dachshund 1 (Drosophila) | 1.441 | 0.049 |
| 17363082 | *Olfr1437* | olfactory receptor 1437 | 1.440 | 0.011 |
| 17516274 | *Olfr961* | olfactory receptor 961 | 1.437 | 0.036 |
| 17214106 | *Igfbp2* | insulin-like growth factor binding protein 2 | 1.432 | 0.039 |
| 17434813 | *Sema3e* | sema domain, immunoglobulin domain (Ig), short basic domain, secreted, (semaphorin) 3E | 1.426 | 0.019 |
| 17252906 | *Mir22* | microRNA 22 | 1.420 | 0.039 |
| 17535448 | *Zfp185* | zinc finger protein 185 | 1.385 | 0.041 |
| 17427367 | *Cyp2j11* | cytochrome P450, family 2, subfamily j, polypeptide 11 | 1.385 | 0.046 |
| 17493995 | *Il18bp* | interleukin 18 binding protein | 1.376 | 0.024 |
| 17487952 | *Ceacam1* | carcinoembryonic antigen-related cell adhesion molecule 1 | 1.375 | 0.030 |
| 17520171 | *Bcl2a1b* | B cell leukemia/lymphoma 2 related protein A1b | 1.374 | 0.019 |
| 17227764 | *Rgs2* | regulator of G-protein signaling 2 | 1.374 | 0.019 |
| 17287160 | *Aspn* | asporin | 1.373 | 0.048 |
| 17548283 | *H3f3b* | H3 histone, family 3B | 1.372 | 0.037 |
| 17253258 | *Coro6* | coronin 6 | 1.369 | 0.013 |
| 17287984 | *Dapk1* | death associated protein kinase 1 | 1.360 | 0.012 |
| 17436235 | *Gm24436* | predicted gene, 24436 | 1.353 | 0.038 |
| 17535183 | *Slitrk2* | SLIT and NTRK-like family, member 2 | 1.353 | 0.027 |
| 17212174 | *Il1r2* | interleukin 1 receptor, type II | 1.352 | 0.015 |
| 17313037 | *n-R5s40* | nuclear encoded rRNA 5S 40 | 1.346 | 0.046 |
| 17463909 | *Mgst1* | microsomal glutathione S-transferase 1 | 1.344 | 0.028 |
| 17492314 | *Mfge8* | milk fat globule-EGF factor 8 protein | 1.342 | 0.021 |
| 17283096 | *Galc* | galactosylceramidase | 1.338 | 0.021 |
| 17216402 | *Serpinb5* | serine (or cysteine) peptidase inhibitor, clade B, member 5 | 1.338 | 0.024 |
| 17392848 | *Rad21l* | RAD21-like (S. pombe) | 1.331 | 0.023 |
| 17272095 | *H3f3b* | H3 histone, family 3B | 1.329 | 0.026 |
| 17395302 | *LOC628110* | uncharacterized LOC628110 | 1.326 | 0.021 |
| 17229537 | *Nos1ap* | nitric oxide synthase 1 (neuronal) adaptor protein | 1.318 | 0.047 |
| 17413852 | *Galnt12* | UDP-N-acetyl-alpha-D-galactosamine:polypeptide N-acetylgalactosaminyltransferase 12 | 1.317 | 0.030 |
| 17354282 | *Cdo1* | cysteine dioxygenase 1, cytosolic | 1.315 | 0.004 |
| 17332728 | *Cldn20* | claudin 20 | 1.311 | 0.048 |
| 17272509 | *Mxra7* | matrix-remodelling associated 7 | 1.310 | 0.035 |
| 17255759 | *LOC102632994* | uncharacterized LOC102632994 | 1.309 | 0.004 |
| 17516408 | *Mir100* | microRNA 100 | 1.306 | 0.036 |
| 17250849 | *B430319H21Rik* | RIKEN cDNA B430319H21 gene | 1.305 | 0.042 |
| 17354784 | *Mir143hg* | Mir143 and Mir145 host gene (non-coding RNA) | 1.304 | 0.039 |
| 17239142 | *Samd5* | sterile alpha motif domain containing 5 | 1.303 | 0.025 |
| 17227401 | *Igfn1* | immunoglobulin-like and fibronectin type III domain containing 1 | 1.301 | 0.027 |
| 17273948 | *Sdc1* | syndecan 1 | 1.295 | 0.045 |
| 17298874 | *Grid1* | glutamate receptor, ionotropic, delta 1 | 1.295 | 0.027 |
| 17505283 | *Wwp2* | WW domain containing E3 ubiquitin protein ligase 2 | 1.293 | 0.027 |
| 17308772 | *Lacc1* | laccase (multicopper oxidoreductase) domain containing 1 | 1.293 | 0.007 |
| 17358777 | *Stambpl1* | STAM binding protein like 1 | 1.292 | 0.031 |
| 17300058 | *Trav4d-4* | T cell receptor alpha variable 4D-4 | 1.292 | 0.027 |
| 17292598 | *Diras2* | DIRAS family, GTP-binding RAS-like 2 | 1.291 | 0.046 |
| 17501569 | *Nat2* | N-acetyltransferase 2 (arylamine N-acetyltransferase) | 1.282 | 0.034 |
| 17403866 | *Ptger3* | prostaglandin E receptor 3 (subtype EP3) | 1.282 | 0.035 |
| 17407850 | *Ecm1* | extracellular matrix protein 1 | 1.280 | 0.001 |
| 17458924 | *Vmn1r4* | vomeronasal 1 receptor 4 | 1.276 | 0.029 |
| 17388733 | *Cd44* | CD44 antigen | 1.275 | 0.024 |
| 17483061 | *Ccdc101* | coiled-coil domain containing 101 | 1.273 | 0.035 |
| 17310072 | *Gdnf* | glial cell line derived neurotrophic factor | 1.272 | 0.028 |
| 17480030 | *Rab38* | RAB38, member RAS oncogene family | 1.272 | 0.031 |
| 17339529 | *Clip4* | CAP-GLY domain containing linker protein family, member 4 | 1.272 | 0.036 |
| 17225138 | *Htr2b* | 5-hydroxytryptamine (serotonin) receptor 2B | 1.271 | 0.017 |
| 17293706 | *Ctsl* | cathepsin L | 1.268 | 0.034 |
| 17367278 | *Commd3* | COMM domain containing 3 | 1.262 | 0.048 |
| 17357042 | *1700105P06Rik* | RIKEN cDNA 1700105P06 gene | 1.260 | 0.016 |
| 17337741 | *Tnfrsf21* | tumor necrosis factor receptor superfamily, member 21 | 1.259 | 0.007 |
| 17295259 | *1700029F12Rik* | RIKEN cDNA 1700029F12 gene | 1.258 | 0.022 |
| 17347160 | *D630014O11Rik* | RIKEN cDNA D630014O11 gene | 1.258 | 0.049 |
| 17294289 | *Nkd2* | naked cuticle 2 homolog (Drosophila) | 1.257 | 0.045 |
| 17362953 | *Ms4a7* | membrane-spanning 4-domains, subfamily A, member 7 | 1.256 | 0.044 |
| 17522842 | *LOC102635428* | uncharacterized LOC102635428 | 1.256 | 0.032 |
| 17446220 | *Asb10* | ankyrin repeat and SOCS box-containing 10 | 1.255 | 0.024 |
| 17420016 | *Id3* | inhibitor of DNA binding 3 | 1.254 | 0.034 |
| 17464492 | *Mettl20* | methyltransferase like 20 | 1.254 | 0.004 |
| 17404174 | *1810022K09Rik* | RIKEN cDNA 1810022K09 gene | 1.254 | 0.033 |
| 17491701 | *A230057D06Rik* | RIKEN cDNA A230057D06 gene | 1.252 | 0.023 |
| 17338982 | *Vav1* | vav 1 oncogene | 1.251 | 0.015 |
| 17430037 | *Tekt2* | tektin 2 | 1.249 | 0.040 |
| 17370487 | *Olfml2a* | olfactomedin-like 2A | 1.248 | 0.032 |
| 17357810 | *Mpeg1* | macrophage expressed gene 1 | 1.247 | 0.039 |
| 17442149 | *P2rx4* | purinergic receptor P2X, ligand-gated ion channel 4 | 1.246 | 0.022 |
| 17298262 | *Mustn1* | musculoskeletal, embryonic nuclear protein 1 | 1.246 | 0.043 |
| 17335506 | *Pi16* | peptidase inhibitor 16 | 1.244 | 0.046 |
| 17329906 | *Iqcg* | IQ motif containing G | 1.241 | 0.014 |
| 17448444 | *Uchl1os* | ubiquitin carboxy-terminal hydrolase L1, opposite strand | 1.239 | 0.014 |
| 17314840 | *Aqp5* | aquaporin 5 | 1.239 | 0.027 |
| 17326640 | *Rbm11* | RNA binding motif protein 11 | 1.237 | 0.048 |
| 17481598 | *Olfr476* | olfactory receptor 476 | 1.237 | 0.037 |
| 17428655 | *C530005A16Rik* | RIKEN cDNA C530005A16 gene | 1.237 | 0.014 |
| 17533446 | *Gpr34* | G protein-coupled receptor 34 | 1.237 | 0.010 |
| 17251222 | *Gas7* | growth arrest specific 7 | 1.237 | 0.023 |
| 17298631 | *Vstm4* | V-set and transmembrane domain containing 4 | 1.236 | 0.009 |
| 17453179 | *Asl* | argininosuccinate lyase | 1.235 | 0.030 |
| 17227077 | *Prelp* | proline arginine-rich end leucine-rich repeat | 1.234 | 0.046 |
| 17225624 | *5033417F24Rik* | RIKEN cDNA 5033417F24 gene | 1.231 | 0.042 |
| 17291509 | *2610307P16Rik* | RIKEN cDNA 2610307P16 gene | 1.230 | 0.035 |
| 17472705 | *Casc1* | cancer susceptibility candidate 1 | 1.228 | 0.042 |
| 17389034 | *Rcn1* | reticulocalbin 1 | 1.228 | 0.046 |
| 17523481 | *LOC102639251* | zinc finger protein 660-like | 1.227 | 0.023 |
| 17502260 | *Fam129c* | family with sequence similarity 129, member C | 1.223 | 0.032 |
| 17509697 | *Tma16* | translation machinery associated 16 homolog (S. cerevisiae) | 1.222 | 0.047 |
| 17283445 | *Lgmn* | legumain | 1.222 | 0.022 |
| 17496863 | *9130023H24Rik* | RIKEN cDNA 9130023H24 gene | 1.218 | 0.019 |
| 17432783 | *Zfp933* | zinc finger protein 933 | 1.216 | 0.009 |
| 17526776 | *Ncam1* | neural cell adhesion molecule 1 | 1.215 | 0.047 |
| 17538983 | *Foxr2* | forkhead box R2 | 1.215 | 0.031 |
| 17311649 |  | --- | 1.214 | 0.045 |
| 17350243 | *Spink6* | serine peptidase inhibitor, Kazal type 6 | 1.213 | 0.046 |
| 17436099 | *Krtcap3* | keratinocyte associated protein 3 | 1.213 | 0.009 |
| 17501600 | *1700125H03Rik* | RIKEN cDNA 1700125H03 gene | 1.211 | 0.030 |
| 17229113 | *Gorab* | golgin, RAB6-interacting | 1.210 | 0.041 |
| 17377940 | *Asxl1* | additional sex combs like 1 | 1.208 | 0.034 |
| 17442780 | *Glt1d1* | glycosyltransferase 1 domain containing 1 | 1.208 | 0.017 |
| 17415136 | *Adamtsl1* | ADAMTS-like 1 | 1.208 | 0.041 |
| 17399828 | *Pglyrp4* | peptidoglycan recognition protein 4 | 1.204 | 0.037 |
| 17517222 | *Arhgap20* | Rho GTPase activating protein 20 | 1.202 | 0.049 |
| 17293961 | *Zfp429* | zinc finger protein 429 | 0.866 | 0.036 |
| 17325533 | *Lrrc58* | leucine rich repeat containing 58 | 0.865 | 0.042 |
| 17514131 | *Exoc8* | exocyst complex component 8 | 0.865 | 0.016 |
| 17253096 | *Crk* | v-crk sarcoma virus CT10 oncogene homolog (avian) | 0.865 | 0.023 |
| 17255064 | *Mmd* | monocyte to macrophage differentiation-associated | 0.864 | 0.022 |
| 17419840 | *Runx3* | runt related transcription factor 3 | 0.863 | 0.021 |
| 17407418 | *Ivl* | involucrin | 0.862 | 0.037 |
| 17275935 | *4930512B01Rik* | RIKEN cDNA 4930512B01 gene | 0.862 | 0.013 |
| 17415728 | *Angptl3* | angiopoietin-like 3 | 0.862 | 0.021 |
| 17430503 | *Kpna6* | karyopherin (importin) alpha 6 | 0.861 | 0.043 |
| 17223490 | *Tmem237* | transmembrane protein 237 | 0.861 | 0.048 |
| 17428032 | *Zyg11b* | zyg-ll family member B, cell cycle regulator | 0.860 | 0.033 |
| 17323503 | *Lztr1* | leucine-zipper-like transcriptional regulator, 1 | 0.860 | 0.033 |
| 17339824 | *Vit* | vitrin | 0.859 | 0.004 |
| 17408662 | *Hipk1* | homeodomain interacting protein kinase 1 | 0.858 | 0.049 |
| 17445699 | *4930519H02Rik* | RIKEN cDNA 4930519H02 gene | 0.858 | 0.034 |
| 17491777 | *4930554H23Rik* | RIKEN cDNA 4930554H23 gene | 0.857 | 0.049 |
| 17454033 | *Epo* | erythropoietin | 0.857 | 0.022 |
| 17372392 | *Dnajc10* | DnaJ (Hsp40) homolog, subfamily C, member 10 | 0.857 | 0.030 |
| 17448411 | *1700126H18Rik* | RIKEN cDNA 1700126H18 gene | 0.857 | 0.039 |
| 17329926 | *1700007L15Rik* | RIKEN cDNA 1700007L15 gene | 0.856 | 0.022 |
| 17517619 | *Ptpn9* | protein tyrosine phosphatase, non-receptor type 9 | 0.854 | 0.035 |
| 17505826 | *Mon1b* | MON1 homolog b (yeast) | 0.853 | 0.020 |
| 17229939 | *Fcrl6* | Fc receptor-like 6 | 0.852 | 0.049 |
| 17463650 | *2700089E24Rik* | RIKEN cDNA 2700089E24 gene | 0.851 | 0.031 |
| 17394297 | *Pltp* | phospholipid transfer protein | 0.851 | 0.039 |
| 17416110 | *Mier1* | mesoderm induction early response 1 homolog (Xenopus laevis | 0.850 | 0.028 |
| 17401205 | *Syt6* | synaptotagmin VI | 0.850 | 0.025 |
| 17270369 | *Kif18b* | kinesin family member 18B | 0.849 | 0.041 |
| 17412001 | *A530072M11Rik* | RIKEN cDNA gene A530072M11 | 0.849 | 0.038 |
| 17348833 | *Ttr* | transthyretin | 0.848 | 0.003 |
| 17319501 | *Rangap1* | RAN GTPase activating protein 1 | 0.848 | 0.036 |
| 17225146 | *LOC102634967* | uncharacterized LOC102634967 | 0.848 | 0.009 |
| 17405075 | *Pcdh18* | protocadherin 18 | 0.848 | 0.045 |
| 17488848 | *n-R5s153* | nuclear encoded rRNA 5S 153 | 0.847 | 0.038 |
| 17350281 | *Dcp2* | DCP2 decapping enzyme homolog (S. cerevisiae) | 0.847 | 0.041 |
| 17418581 | *Col8a2* | collagen, type VIII, alpha 2 | 0.847 | 0.043 |
| 17342496 | *Rab40c* | Rab40C, member RAS oncogene family | 0.847 | 0.019 |
| 17548042 | *Galnt1* | UDP-N-acetyl-alpha-D-galactosamine:polypeptide N-acetylgalactosaminyltransferase 1 | 0.846 | 0.028 |
| 17390810 | *Gatm* | glycine amidinotransferase (L-arginine:glycine amidinotransferase) | 0.846 | 0.031 |
| 17464704 | *Dlx6as2* | Dlx6 antisense RNA 2 | 0.846 | 0.032 |
| 17358486 | *Cdc37l1* | cell division cycle 37-like 1 | 0.845 | 0.025 |
| 17408497 | *Cd2* | CD2 antigen | 0.845 | 0.035 |
| 17248793 | *Fam71b* | family with sequence similarity 71, member B | 0.845 | 0.023 |
| 17547892 | *Zbed4* | zinc finger, BED domain containing 4 | 0.845 | 0.048 |
| 17235556 | *Zbtb7a* | zinc finger and BTB domain containing 7a | 0.844 | 0.011 |
| 17352997 | *Trappc8* | trafficking protein particle complex 8 | 0.844 | 0.019 |
| 17435622 | *9530036O11Rik* | RIKEN cDNA 9530036O11Rik | 0.844 | 0.004 |
| 17350824 | *Slc12a2* | solute carrier family 12, member 2 | 0.844 | 0.041 |
| 17520830 | *Ephb1* | Eph receptor B1 | 0.843 | 0.041 |
| 17271968 | *Mif4gd* | MIF4G domain containing | 0.843 | 0.047 |
| 17487500 | *Igsf23* | immunoglobulin superfamily, member 23 | 0.842 | 0.019 |
| 17300151 | *Traj51* | T cell receptor alpha joining 51 | 0.841 | 0.039 |
| 17512572 | *Slc12a4* | solute carrier family 12, member 4 | 0.841 | 0.034 |
| 17292190 | *Nup153* | nucleoporin 153 | 0.841 | 0.029 |
| 17398272 | *Arl14* | ADP-ribosylation factor-like 14 | 0.841 | 0.050 |
| 17453935 | *Zan* | zonadhesin | 0.841 | 0.028 |
| 17319707 | *Cyp2d26* | cytochrome P450, family 2, subfamily d, polypeptide 26 | 0.841 | 0.020 |
| 17388128 | *Madd* | MAP-kinase activating death domain | 0.840 | 0.028 |
| 17520716 | *1600029I14Rik* | RIKEN cDNA 1600029I14 gene | 0.840 | 0.040 |
| 17229988 | *Cadm3* | cell adhesion molecule 3 | 0.840 | 0.034 |
| 17399840 |  | --- | 0.839 | 0.008 |
| 17240737 | *Gprc6a* | G protein-coupled receptor, family C, group 6, member A | 0.839 | 0.030 |
| 17261098 | *Pex13* | peroxisomal biogenesis factor 13 | 0.839 | 0.046 |
| 17525578 | *Slc37a2* | solute carrier family 37 (glycerol-3-phosphate transporter), member 2 | 0.837 | 0.025 |
| 17453557 | *Pom121* | nuclear pore membrane protein 121 | 0.836 | 0.024 |
| 17408182 | *Olfr1402* | olfactory receptor 1402 | 0.836 | 0.031 |
| 17470445 | *Atp6v1e1* | ATPase, H+ transporting, lysosomal V1 subunit E1 | 0.836 | 0.033 |
| 17320796 | *Twf1* | twinfilin, actin-binding protein, homolog 1 (Drosophila) | 0.835 | 0.021 |
| 17326650 | *LOC102632240* | uncharacterized LOC102632240 | 0.834 | 0.031 |
| 17529058 | *Gm17324* | predicted gene, 17324 | 0.834 | 0.040 |
| 17244937 | *4933440J02Rik* | RIKEN cDNA 4933440J02 gene | 0.833 | 0.034 |
| 17347650 | *Haao* | 3-hydroxyanthranilate 3,4-dioxygenase | 0.833 | 0.002 |
| 17505239 | *Nip7* | nuclear import 7 homolog (S. cerevisiae) | 0.832 | 0.027 |
| 17311473 | *Aard* | alanine and arginine rich domain containing protein | 0.832 | 0.031 |
| 17337566 | *Olfr107* | olfactory receptor 107 | 0.832 | 0.021 |
| 17266911 | *Mmp28* | matrix metallopeptidase 28 (epilysin) | 0.832 | 0.033 |
| 17241474 | *Stox1* | storkhead box 1 | 0.831 | 0.011 |
| 17280054 | *Trib2* | tribbles homolog 2 (Drosophila) | 0.831 | 0.044 |
| 17509617 | *Cpe* | carboxypeptidase E | 0.828 | 0.027 |
| 17363779 | *Ak3* | adenylate kinase 3 | 0.827 | 0.048 |
| 17334449 | *Hagh* | hydroxyacyl glutathione hydrolase | 0.827 | 0.026 |
| 17494087 | *Olfr543* | olfactory receptor 543 | 0.827 | 0.031 |
| 17314848 | *Aqp6* | aquaporin 6 | 0.827 | 0.024 |
| 17432687 | *Tnfrsf8* | tumor necrosis factor receptor superfamily, member 8 | 0.826 | 0.030 |
| 17284948 | *Akr1c19* | aldo-keto reductase family 1, member C19 | 0.826 | 0.047 |
| 17334716 | *Gng13* | guanine nucleotide binding protein (G protein), gamma 13 | 0.826 | 0.007 |
| 17238687 | *Olfr804* | olfactory receptor 804 | 0.826 | 0.043 |
| 17266732 | *Asic2* | acid-sensing (proton-gated) ion channel 2 | 0.826 | 0.024 |
| 17274670 | *Myt1l* | myelin transcription factor 1-like | 0.825 | 0.040 |
| 17535033 | *Vgll1* | vestigial like 1 homolog (Drosophila) | 0.823 | 0.025 |
| 17474278 | *Ceacam5* | carcinoembryonic antigen-related cell adhesion molecule 5 | 0.822 | 0.040 |
| 17323297 | *Cebpd* | CCAAT/enhancer binding protein (C/EBP), delta | 0.821 | 0.028 |
| 17251933 | *Asgr1* | asialoglycoprotein receptor 1 | 0.821 | 0.042 |
| 17409621 | *S1pr1* | sphingosine-1-phosphate receptor 1 | 0.819 | 0.043 |
| 17463482 | *Klrb1f* | killer cell lectin-like receptor subfamily B member 1F | 0.819 | 0.040 |
| 17350966 | *G630071F17Rik* | RIKEN cDNA G630071F17 gene | 0.819 | 0.050 |
| 17321326 | *Arf3* | ADP-ribosylation factor 3 | 0.818 | 0.040 |
| 17334275 | *Caskin1* | CASK interacting protein 1 | 0.817 | 0.020 |
| 17344852 | *Olfr97* | olfactory receptor 97 | 0.817 | 0.017 |
| 17328978 | *Cdc45* | cell division cycle 45 | 0.814 | 0.036 |
| 17314910 | *Larp4* | La ribonucleoprotein domain family, member 4 | 0.814 | 0.036 |
| 17483772 | *Sec23ip* | Sec23 interacting protein | 0.814 | 0.044 |
| 17335493 | *BC004004* | cDNA sequence BC004004 | 0.813 | 0.019 |
| 17379395 | *Svs5* | seminal vesicle secretory protein 5 | 0.812 | 0.014 |
| 17520624 | *Rbp1* | retinol binding protein 1, cellular | 0.812 | 0.025 |
| 17317056 | *Enpp2* | ectonucleotide pyrophosphatase/phosphodiesterase 2 | 0.812 | 0.008 |
| 17479012 | *Lins* | lines homolog (Drosophila) | 0.812 | 0.027 |
| 17347839 | *Six2* | sine oculis-related homeobox 2 | 0.812 | 0.009 |
| 17411420 | *Cth* | cystathionase (cystathionine gamma-lyase) | 0.811 | 0.047 |
| 17402305 | *Bcar3* | breast cancer anti-estrogen resistance 3 | 0.811 | 0.017 |
| 17319982 | *1810041L15Rik* | RIKEN cDNA 1810041L15 gene | 0.809 | 0.044 |
| 17493807 | *Plekhb1* | pleckstrin homology domain containing, family B (evectins) member 1 | 0.808 | 0.044 |
| 17498892 | *Abhd13* | abhydrolase domain containing 13 | 0.807 | 0.040 |
| 17281291 | *E030019B13Rik* | RIKEN cDNA E030019B13 gene | 0.807 | 0.039 |
| 17537118 | *P2ry10* | purinergic receptor P2Y, G-protein coupled 10 | 0.807 | 0.008 |
| 17347457 | *Atl2* | atlastin GTPase 2 | 0.804 | 0.045 |
| 17254071 | *Tmem132e* | transmembrane protein 132E | 0.804 | 0.021 |
| 17523650 | *Ccr2* | chemokine (C-C motif) receptor 2 | 0.804 | 0.000 |
| 17320993 | *Endou* | endonuclease, polyU-specific | 0.803 | 0.019 |
| 17489571 | *Scgb2b27* | secretoglobin, family 2B, member 27 | 0.803 | 0.031 |
| 17258178 | *Cdr2l* | cerebellar degeneration-related protein 2-like | 0.803 | 0.027 |
| 17308556 | *Cysltr2* | cysteinyl leukotriene receptor 2 | 0.802 | 0.016 |
| 17403934 | *Rpe65* | retinal pigment epithelium 65 | 0.801 | 0.029 |
| 17277130 | *2410016O06Rik* | RIKEN cDNA 2410016O06 gene | 0.800 | 0.043 |
| 17459097 | *Mmrn1* | multimerin 1 | 0.800 | 0.035 |
| 17429341 | *AU022252* | expressed sequence AU022252 | 0.799 | 0.042 |
| 17543246 | *4932442L08Rik* | RIKEN cDNA 4932442L08 gene | 0.799 | 0.007 |
| 17217891 | *4933436E23Rik* | RIKEN cDNA 4933436E23 gene | 0.798 | 0.016 |
| 17378860 | *Ralgapb* | Ral GTPase activating protein, beta subunit (non-catalytic) | 0.798 | 0.006 |
| 17266498 | *Nlk* | nemo like kinase | 0.798 | 0.040 |
| 17537993 | *Fam199x* | family with sequence similarity 199, X-linked | 0.797 | 0.012 |
| 17294246 | *D630045M09Rik* | RIKEN cDNA D630045M09 gene | 0.797 | 0.036 |
| 17292698 | *4833439L19Rik* | RIKEN cDNA 4833439L19 gene | 0.797 | 0.029 |
| 17287115 | *Bicd2* | bicaudal D homolog 2 (Drosophila) | 0.796 | 0.042 |
| 17462451 | *Slc6a13* | solute carrier family 6 (neurotransmitter transporter, GABA), member 13 | 0.795 | 0.029 |
| 17477116 | *Etfb* | electron transferring flavoprotein, beta polypeptide | 0.795 | 0.044 |
| 17510311 | *Abhd8* | abhydrolase domain containing 8 | 0.795 | 0.046 |
| 17507355 | *Lig4* | ligase IV, DNA, ATP-dependent | 0.795 | 0.036 |
| 17217844 | *Mir181a-1* | microRNA 181a-1 | 0.794 | 0.023 |
| 17326917 | *2310034C09Rik* | RIKEN cDNA 2310034C09 gene | 0.793 | 0.040 |
| 17542149 | *Ids* | iduronate 2-sulfatase | 0.793 | 0.044 |
| 17539927 | *Eras* | ES cell-expressed Ras | 0.791 | 0.020 |
| 17237170 | *Pawr* | PRKC, apoptosis, WT1, regulator | 0.790 | 0.013 |
| 17318045 | *Cyp11b1* | cytochrome P450, family 11, subfamily b, polypeptide 1 | 0.790 | 0.018 |
| 17462694 | *Nanog* | Nanog homeobox | 0.789 | 0.032 |
| 17255534 | *Hoxb13* | homeobox B13 | 0.788 | 0.039 |
| 17264440 | *Odf4* | outer dense fiber of sperm tails 4 | 0.785 | 0.035 |
| 17351681 | *Mex3c* | mex3 homolog C (C. elegans) | 0.785 | 0.031 |
| 17508416 | *Bag4* | BCL2-associated athanogene 4 | 0.785 | 0.032 |
| 17305243 | *Sftpd* | surfactant associated protein D | 0.784 | 0.017 |
| 17229020 | *Fmo1* | flavin containing monooxygenase 1 | 0.784 | 0.023 |
| 17426497 | *Megf9* | multiple EGF-like-domains 9 | 0.784 | 0.044 |
| 17341625 | *Tceb2* | transcription elongation factor B (SIII), polypeptide 2 | 0.784 | 0.013 |
| 17340215 | *Tmem247* | transmembrane protein 247 | 0.784 | 0.006 |
| 17509506 | *Clcn3* | chloride channel 3 | 0.782 | 0.022 |
| 17517424 | *Ireb2* | iron responsive element binding protein 2 | 0.782 | 0.045 |
| 17480652 | *Pgm2l1* | phosphoglucomutase 2-like 1 | 0.782 | 0.018 |
| 17250178 | *Hist3h2a* | histone cluster 3, H2a | 0.782 | 0.033 |
| 17410636 | *Mttp* | microsomal triglyceride transfer protein | 0.781 | 0.024 |
| 17222951 | *1700019D03Rik* | RIKEN cDNA 1700019D03 gene | 0.780 | 0.021 |
| 17400862 | *Hmgcs2* | 3-hydroxy-3-methylglutaryl-Coenzyme A synthase 2 | 0.780 | 0.028 |
| 17486128 | *Zim3* | zinc finger, imprinted 3 | 0.779 | 0.047 |
| 17445525 | *Rundc3b* | RUN domain containing 3B | 0.778 | 0.024 |
| 17323587 | *Rpl26-ps4* | ribosomal protein L26, pseudogene 4 | 0.778 | 0.010 |
| 17411216 | *Fam73a* | family with sequence similarity 73, member A | 0.777 | 0.009 |
| 17289137 | *Homer1* | homer homolog 1 (Drosophila) | 0.777 | 0.017 |
| 17246231 | *Erbb3* | v-erb-b2 erythroblastic leukemia viral oncogene homolog 3 (avian) | 0.777 | 0.041 |
| 17459482 | *Cd8a* | CD8 antigen, alpha chain | 0.776 | 0.048 |
| 17410410 | *Sgms2* | sphingomyelin synthase 2 | 0.776 | 0.003 |
| 17233799 | *Slc25a16* | solute carrier family 25 (mitochondrial carrier, Graves disease autoantigen), member 16 | 0.774 | 0.021 |
| 17288354 | *BC048507* | cDNA sequence BC048507 | 0.774 | 0.035 |
| 17288338 | *Zfp493* | zinc finger protein 493 | 0.774 | 0.044 |
| 17344200 | *Ly6g6d* | lymphocyte antigen 6 complex, locus G6D | 0.773 | 0.038 |
| 17494148 | *Olfr573-ps1* | olfactory receptor 573, pseudogene 1 | 0.773 | 0.006 |
| 17218889 | *Slc19a2* | solute carrier family 19 (thiamine transporter), member 2 | 0.772 | 0.049 |
| 17334253 | *Eci1* | enoyl-Coenzyme A delta isomerase 1 | 0.772 | 0.046 |
| 17518628 | *Oaz2* | ornithine decarboxylase antizyme 2 | 0.772 | 0.013 |
| 17415219 | *Acer2* | alkaline ceramidase 2 | 0.772 | 0.049 |
| 17361299 | *2010003K11Rik* | RIKEN cDNA 2010003K11 gene | 0.771 | 0.041 |
| 17527421 | *Tspan3* | tetraspanin 3 | 0.770 | 0.029 |
| 17232937 | *Ostm1* | osteopetrosis associated transmembrane protein 1 | 0.770 | 0.006 |
| 17259937 | *Sec14l2* | SEC14-like 2 (S. cerevisiae) | 0.770 | 0.038 |
| 17430103 | *Ago4* | argonaute RISC catalytic subunit 4 | 0.770 | 0.038 |
| 17267115 | *Usp32* | ubiquitin specific peptidase 32 | 0.770 | 0.033 |
| 17460760 | *Vmn1r50* | vomeronasal 1 receptor 50 | 0.769 | 0.030 |
| 17520307 | *Plscr5* | phospholipid scramblase family, member 5 | 0.768 | 0.018 |
| 17319450 | *8430426J06Rik* | RIKEN cDNA 8430426J06 gene | 0.767 | 0.022 |
| 17376538 | *4930425F17Rik* | RIKEN cDNA 4930425F17 gene | 0.767 | 0.018 |
| 17348113 | *Lyzl1* | lysozyme-like 1 | 0.766 | 0.033 |
| 17219764 | *Olfr220* | olfactory receptor 220 | 0.764 | 0.029 |
| 17483420 | *Ctf1* | cardiotrophin 1 | 0.763 | 0.018 |
| 17470301 | *Adipor2* | adiponectin receptor 2 | 0.763 | 0.039 |
| 17395920 | *Samd10* | sterile alpha motif domain containing 10 | 0.763 | 0.037 |
| 17517848 | *Stra6* | stimulated by retinoic acid gene 6 | 0.762 | 0.033 |
| 17253024 | *Pitpna* | phosphatidylinositol transfer protein, alpha | 0.762 | 0.020 |
| 17394707 | *Spata2* | spermatogenesis associated 2 | 0.761 | 0.021 |
| 17407956 | *Car14* | carbonic anhydrase 14 | 0.760 | 0.003 |
| 17495466 | *Arl6ip1* | ADP-ribosylation factor-like 6 interacting protein 1 | 0.760 | 0.024 |
| 17508170 | *1810011O10Rik* | RIKEN cDNA 1810011O10 gene | 0.760 | 0.019 |
| 17337966 | *F630040K05Rik* | RIKEN cDNA F630040K05 gene | 0.759 | 0.010 |
| 17320100 | *Pkdrej* | polycystic kidney disease (polycystin) and REJ (sperm receptor for egg jelly homolog, sea urchin) | 0.759 | 0.049 |
| 17275572 | *Insm2* | insulinoma-associated 2 | 0.758 | 0.008 |
| 17335983 | *Cyp4f37* | cytochrome P450, family 4, subfamily f, polypeptide 37 | 0.758 | 0.045 |
| 17433537 | *BC039966* | cDNA sequence BC039966 | 0.757 | 0.036 |
| 17294215 | *Irx1* | Iroquois related homeobox 1 (Drosophila) | 0.757 | 0.033 |
| 17309340 | *Spry2* | sprouty homolog 2 (Drosophila) | 0.756 | 0.047 |
| 17456176 | *Met* | met proto-oncogene | 0.754 | 0.038 |
| 17405549 | *Plch1* | phospholipase C, eta 1 | 0.754 | 0.047 |
| 17324714 | *Pigz* | phosphatidylinositol glycan anchor biosynthesis, class Z | 0.752 | 0.033 |
| 17471201 | *Rad51ap1* | RAD51 associated protein 1 | 0.751 | 0.040 |
| 17525894 | *Sorl1* | sortilin-related receptor, LDLR class A repeats-containing | 0.750 | 0.039 |
| 17478864 | *Mtmr10* | myotubularin related protein 10 | 0.749 | 0.030 |
| 17274021 | *Osr1* | odd-skipped related 1 (Drosophila) | 0.748 | 0.029 |
| 17385478 | *5330411J11Rik* | RIKEN cDNA 5330411J11 gene | 0.747 | 0.018 |
| 17398416 | *Pdgfc* | platelet-derived growth factor, C polypeptide | 0.747 | 0.036 |
| 17292058 | *Elovl2* | elongation of very long chain fatty acids (FEN1/Elo2, SUR4/Elo3, yeast)-like 2 | 0.747 | 0.019 |
| 17498730 | *Mcemp1* | mast cell expressed membrane protein 1 | 0.744 | 0.009 |
| 17319009 | *Il2rb* | interleukin 2 receptor, beta chain | 0.743 | 0.039 |
| 17339434 | *Lpin2* | lipin 2 | 0.743 | 0.044 |
| 17464850 | *Thsd7a* | thrombospondin, type I, domain containing 7A | 0.743 | 0.040 |
| 17362527 | *Rom1* | rod outer segment membrane protein 1 | 0.742 | 0.002 |
| 17328046 | *Grin2a* | glutamate receptor, ionotropic, NMDA2A (epsilon 1) | 0.741 | 0.015 |
| 17369821 | *Ptges2* | prostaglandin E synthase 2 | 0.740 | 0.029 |
| 17341589 | *Paqr4* | progestin and adipoQ receptor family member IV | 0.739 | 0.025 |
| 17253996 | *Tmem98* | transmembrane protein 98 | 0.735 | 0.047 |
| 17211043 | *Sgk3* | serum/glucocorticoid regulated kinase 3 | 0.733 | 0.026 |
| 17423395 | *Slc26a7* | solute carrier family 26, member 7 | 0.733 | 0.016 |
| 17246352 | *Gdf11* | growth differentiation factor 11 | 0.732 | 0.024 |
| 17347267 | *Fez2* | fasciculation and elongation protein zeta 2 (zygin II) | 0.731 | 0.033 |
| 17521014 | *Acad11* | acyl-Coenzyme A dehydrogenase family, member 11 | 0.731 | 0.025 |
| 17231522 | *Lrp11* | low density lipoprotein receptor-related protein 11 | 0.731 | 0.033 |
| 17234917 | *Gzmm* | granzyme M (lymphocyte met-ase 1) | 0.730 | 0.048 |
| 17294458 | *Rhobtb3* | Rho-related BTB domain containing 3 | 0.730 | 0.000 |
| 17360695 | *Atrnl1* | attractin like 1 | 0.729 | 0.002 |
| 17523416 | *Abhd5* | abhydrolase domain containing 5 | 0.728 | 0.005 |
| 17404373 | *Naaladl2* | N-acetylated alpha-linked acidic dipeptidase-like 2 | 0.728 | 0.039 |
| 17253630 | *Unc119* | unc-119 homolog (C. elegans) | 0.726 | 0.030 |
| 17265792 | *Olfr392* | olfactory receptor 392 | 0.722 | 0.003 |
| 17315743 | *Osmr* | oncostatin M receptor | 0.722 | 0.049 |
| 17448847 | *Usp46* | ubiquitin specific peptidase 46 | 0.722 | 0.025 |
| 17529398 | *Me1* | malic enzyme 1, NADP(+)-dependent, cytosolic | 0.718 | 0.013 |
| 17516145 | *Olfr894* | olfactory receptor 894 | 0.718 | 0.018 |
| 17530243 | *Trf* | transferrin | 0.717 | 0.014 |
| 17266586 | *Omg* | oligodendrocyte myelin glycoprotein | 0.715 | 0.012 |
| 17416540 | *Lrp8* | low density lipoprotein receptor-related protein 8, apolipoprotein e receptor | 0.715 | 0.048 |
| 17409873 | *Tmem56* | transmembrane protein 56 | 0.715 | 0.048 |
| 17528836 | *Mapk6* | mitogen-activated protein kinase 6 | 0.713 | 0.030 |
| 17480036 | *Fzd4* | frizzled homolog 4 (Drosophila) | 0.712 | 0.049 |
| 17449322 | *Ugt2b1* | UDP glucuronosyltransferase 2 family, polypeptide B1 | 0.711 | 0.033 |
| 17254041 | *Ccl2* | chemokine (C-C motif) ligand 2 | 0.709 | 0.046 |
| 17472114 | *Plbd1* | phospholipase B domain containing 1 | 0.709 | 0.012 |
| 17316175 | *Basp1* | brain abundant, membrane attached signal protein 1 | 0.709 | 0.026 |
| 17246358 | *Rdh5* | retinol dehydrogenase 5 | 0.709 | 0.016 |
| 17283014 | *Gtf2a1* | general transcription factor II A, 1 | 0.708 | 0.006 |
| 17513479 | *Tldc1* | TBC/LysM associated domain containing 1 | 0.704 | 0.017 |
| 17463727 | *Apold1* | apolipoprotein L domain containing 1 | 0.702 | 0.006 |
| 17355728 | *Mir5127* | microRNA 5127 | 0.701 | 0.043 |
| 17229036 | *Fmo2* | flavin containing monooxygenase 2 | 0.700 | 0.036 |
| 17414984 | *Lurap1l* | leucine rich adaptor protein 1-like | 0.696 | 0.046 |
| 17245152 | *Rab3ip* | RAB3A interacting protein | 0.695 | 0.037 |
| 17346007 | *Sult1c1* | sulfotransferase family, cytosolic, 1C, member 1 | 0.692 | 0.049 |
| 17409994 |  | --- | 0.691 | 0.044 |
| 17317393 | *Fam84b* | family with sequence similarity 84, member B | 0.687 | 0.028 |
| 17436823 | *Adra2c* | adrenergic receptor, alpha 2c | 0.686 | 0.006 |
| 17316700 | *Azin1* | antizyme inhibitor 1 | 0.685 | 0.017 |
| 17479354 | *Abhd2* | abhydrolase domain containing 2 | 0.684 | 0.022 |
| 17283364 | *Tc2n* | tandem C2 domains, nuclear | 0.683 | 0.047 |
| 17250660 | *Adora2b* | adenosine A2b receptor | 0.682 | 0.021 |
| 17518780 | *Car12* | carbonic anyhydrase 12 | 0.681 | 0.046 |
| 17516303 | *1700001J11Rik* | ring finger protein 19A pseudogene | 0.681 | 0.026 |
| 17329036 | *Olfr168* | olfactory receptor 168 | 0.676 | 0.019 |
| 17211198 | *Sulf1* | sulfatase 1 | 0.676 | 0.008 |
| 17277766 | *Tshr* | thyroid stimulating hormone receptor | 0.675 | 0.045 |
| 17309154 | *Kctd12* | potassium channel tetramerisation domain containing 12 | 0.671 | 0.047 |
| 17503932 | *Mt3* | metallothionein 3 | 0.669 | 0.010 |
| 17495617 | *Gpr139* | G protein-coupled receptor 139 | 0.666 | 0.036 |
| 17267656 | *Nog* | noggin | 0.666 | 0.006 |
| 17487507 | *n-R5s152* | nuclear encoded rRNA 5S 152 | 0.663 | 0.039 |
| 17359160 | *Plce1* | phospholipase C, epsilon 1 | 0.662 | 0.044 |
| 17464614 | *Pon1* | paraoxonase 1 | 0.662 | 0.010 |
| 17326756 | *Ncam2* | neural cell adhesion molecule 2 | 0.660 | 0.038 |
| 17433656 | *5930403L14Rik* | RIKEN cDNA 5930403L14 gene | 0.660 | 0.028 |
| 17217350 | *Etnk2* | ethanolamine kinase 2 | 0.655 | 0.043 |
| 17319142 | *Slc16a8* | solute carrier family 16 (monocarboxylic acid transporters), member 8 | 0.652 | 0.007 |
| 17386210 | *Lrp2* | low density lipoprotein receptor-related protein 2 | 0.652 | 0.023 |
| 17339801 | *Crim1* | cysteine rich transmembrane BMP regulator 1 (chordin like) | 0.651 | 0.038 |
| 17211258 | *Rdh10* | retinol dehydrogenase 10 (all-trans) | 0.651 | 0.007 |
| 17429310 | *Zfp691* | zinc finger protein 691 | 0.645 | 0.017 |
| 17514961 | *Olfr869* | olfactory receptor 869 | 0.645 | 0.026 |
| 17329479 | *Cldn1* | claudin 1 | 0.644 | 0.027 |
| 17401335 | *Slc16a1* | solute carrier family 16 (monocarboxylic acid transporters), member 1 | 0.642 | 0.004 |
| 17410311 | *Rrh* | retinal pigment epithelium derived rhodopsin homolog | 0.641 | 0.005 |
| 17406247 | *Lrat* | lecithin-retinol acyltransferase (phosphatidylcholine-retinol-O-acyltransferase) | 0.638 | 0.031 |
| 17505455 | *Phlpp2* | PH domain and leucine rich repeat protein phosphatase 2 | 0.637 | 0.019 |
| 17531370 | *Fbxw16* | F-box and WD-40 domain protein 16 | 0.637 | 0.015 |
| 17429632 | *Mfsd2a* | major facilitator superfamily domain containing 2A | 0.637 | 0.016 |
| 17364820 | *Sfrp5* | secreted frizzled-related sequence protein 5 | 0.633 | 0.027 |
| 17240556 | *Nr2e1* | nuclear receptor subfamily 2, group E, member 1 | 0.631 | 0.043 |
| 17495821 | *Cdr2* | cerebellar degeneration-related 2 | 0.627 | 0.022 |
| 17284839 | *Itgb8* | integrin beta 8 | 0.626 | 0.039 |
| 17531181 |  | --- | 0.625 | 0.045 |
| 17232119 | *Slc2a12* | solute carrier family 2 (facilitated glucose transporter), member 12 | 0.625 | 0.007 |
| 17402181 | *F3* | coagulation factor III | 0.613 | 0.010 |
| 17291053 | *Hist1h4m* | histone cluster 1, H4m | 0.610 | 0.041 |
| 17372515 | *Itgav* | integrin alpha V | 0.591 | 0.029 |
| 17358466 | *Slc1a1* | solute carrier family 1 (neuronal/epithelial high affinity glutamate transporter, system Xag), member 1 | 0.589 | 0.042 |
| 17318587 | *Slc39a4* | solute carrier family 39 (zinc transporter), member 4 | 0.576 | 0.023 |
| 17305143 | *Cdhr1* | cadherin-related family member 1 | 0.549 | 0.009 |
| 17244973 | *Trhde* | TRH-degrading enzyme | 0.547 | 0.005 |
| 17434973 | *Sema3c* | sema domain, immunoglobulin domain (Ig), short basic domain, secreted, (semaphorin) 3C | 0.537 | 0.036 |
| 17298917 | *Lrit2* | leucine-rich repeat, immunoglobulin-like and transmembrane domains 2 | 0.530 | 0.014 |
| 17477322 | *Klk1b26* | kallikrein 1-related petidase b26 | 0.527 | 0.031 |
| 17236882 | *Dusp6* | dual specificity phosphatase 6 | 0.524 | 0.006 |

| **Supplementary Table S3. Dry AMD-like ultrastructural pathology in dry AMD mouse models.** | | | | | | |
| --- | --- | --- | --- | --- | --- | --- |
| No. | Mouse model | Age  (mo) | Aβ deposits | RPE change | Basal depostis | BM changes |
| 1 | 5XFAD mice | 12 | Aβ | Loss of apical microvilli and basal infolding, loss of tight junctions, increased lipofuscin granules, cystic degeneration | BlamD and BlinD | Thickening |
| 2 | *neprilysin* -/- [19] | 27 | Aβ | Vacuolization, loss of tight and adherens junctions, distorted basal infolding, degeneration | BlamD and BlinD | None |
| 3 | *mcd/mcd* mice [20] | 18 | - | Hypertrophy, hypopigmentation | BlamD and BlinD | Minimal thickening |
| 3 | *Ccl2*-/- [12] | 9 | - | Vacuolated, lipofuscin, menalosomes | Present | Thickening |
| 4 | *Ccr2*-/- [12] | 9 | - | Hypopigmentation, loss of basal infolding | present | Thickening |
| 6 | APOE4 TR [18] | 32 | Aβ | Vacuolization, hyperpigmentation, hypopigmentation, atrophy, disorganized basal infoldings | BlamD | Thickening |
| 7 | *Cfh*+/- [21] | 24 | - | Hypertrophy, increased vescicles | BlamD | Thickening |

BlamD, Basal laminar deposits; BlinD, Basal linear deposits
